# Meta-analysis of Osteopontin splice variants in cancer

**DOI:** 10.1186/s12885-023-10854-x

**Published:** 2023-04-24

**Authors:** Yu An, Gulimirerouzi Fnu, Changchun Xie, Georg F. Weber

**Affiliations:** 1 Division of Biostatistics and Bioinformatics, Department of Environmental and Public Health Sciences, University of Cincinnati Academic Health Center, Cincinnati, OH USA; 2 James L. Winkle College of Pharmacy, College of Pharmacy, University of Cincinnati Academic Health Center, 231 Albert Sabin Way, Cincinnati, OH USA

**Keywords:** Osteopontin, Splice variant, Biomarker, Cancer, Grade, Stage, Prognosis, Survival

## Abstract

**Background:**

The cytokine Osteopontin is a mediator of tumor progression and cancer metastasis. In 2006, we reported that (in addition to the full-length form -a) splice variants of Osteopontin (forms -b and -c) are produced selectively by transformed cells. Through June 2021, 36 PubMed-indexed journal articles have studied Osteopontin splice variants in various cancer patients.

**Methods:**

Applying a categorical approach previously developed by us, here we conduct a meta-analysis of the pertinent literature. We supplement this with evaluation of the relevant entries in the TSVdb database, which focusses on splice variant expression, thus including the additional variants -4 and -5. The analysis covers 5886 patients across 15 tumors from the literature and 10,446 patients across 33 tumors from TSVdb.

**Results:**

The database yields positive results more frequently than the categorical meta-analysis. The two sources are in agreement on the elevation of OPN-a, OPN-b, and OPN-c in lung cancer and the elevation of OPN-c in breast cancer as compared to healthy tissue. Specific splice variants are associated with grade, stage, or patient survival pertaining to various cancers.

**Conclusions:**

There are cases of persisting discrepancies, which require further investigation to clarify the Osteopontin splice variant utilization, so that their diagnostic, prognostic and potentially predictive potential can be brought to fruition.

**Supplementary Information:**

The online version contains supplementary material available at 10.1186/s12885-023-10854-x.

## Introduction

There continues to be a need for clinically applicable biomarkers of cancer progression. A candidate for this use is the metastasis mediator Osteopontin (OPN, secreted phosphoprotein 1, SPP1, gene ID 6696), which is over-expressed in about 30 malignancies [1, 2]. It has been extensively studied but has not found utilization in clinical diagnostics. While total Osteopontin (pan-Osteopontin, covering all variant forms) is associated with stage, grade, and prognosis, it is compromised, not only by lacking specificity for a particular type of cancer, but also by its physiologic role as a type I (Th1) inducer cytokine in the immune system [3]. As such, it is induced by infections with viruses or intracellular pathogens. Another challenge for applying the diagnostic marker Osteopontin in the clinic has been its high variability on the post-translational level. The protein is characterized by substantial glycosylation and phosphorylation, it may be subject to transglutamination, and it avidly binds calcium and heparin; there also is a site for sulfation. Osteopontin can be cleaved by various proteases, generating multiple smaller entities.

**Fig. 1:**
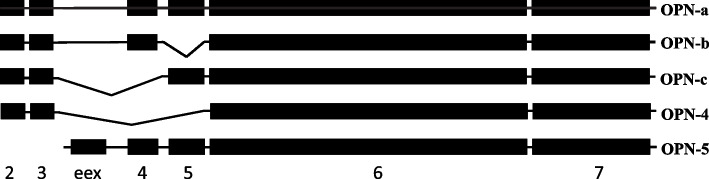
Exon arrangement and splice variants of Osteopontin. Exon 1 in silent (untranslated), the other exons are depicted as black boxes. The full-length form, OPN-a, is depicted in the top row. The alternatively spliced exons 4 and 5 generate the isoforms OPN-b, -c and -4. Uniquely, the form OPN-5 retains an extra exon (eex), located between the canonical exons 3 and 4, which conveys an alternative translation start (due to this, OPN-5 constitutes a larger protein)

The gene products of cancer progression are typically not mutated in transformed cells, but are aberrantly expressed or spliced [4–6]. On the transcript level, Osteopontin can yield five forms, OPN-a (full-length), OPN-b (lacking exon 5), OPN-c (lacking exon 4), OPN-4 (lacking exons 4 and 5), and OPN-5 (alternative N-terminus upstream of exon 4) [7] (Fig. 1). A focus on alternative splicing offers several advantages for diagnostic use. Individual forms are detectable on the RNA level with probes or primers that span the splice junctions. On the protein level, the epitopes that characterize the variants are often free of amino acid decoration. Antibodies have been raised to the amino acid sequence surrounding the OPN-c splice junction [8, 9]. The observation that alternative splice variants of Osteopontin may be produced selectively in cancer, and that distinct types of cancer may express different combinations of spice variants has opened the field for a more refined evaluation of the potential for utilizing Osteopontin forms in cancer detection, assessment of aggressiveness, prognostication, or prediction of treatment responses. In sum, many of the challenges experienced by the biomarker pan-Osteopontin do not pose concerns when individual splice variants are employed as indicators instead.

Since the original report of Osteopontin splicing in cancer [10], a body of publications has been produced that have investigated splice variant expression in tumor specimens from patients. The accrued knowledge base justifies a meta-analysis to elucidate, which Osteopontin variants are associated with specific types of cancer, whether they increase with grade or stage, and what value they may have for prognostication. This is important for realizing the clinical potential. The algorithms of categorical meta-analysis [1, 2] have proven successful in extracting relevant information from the noise of varying reporting methods. They are applied here for evaluation of the literature. Furthermore, we include an investigation of entries in the TCGA Splicing Variants database TSVdb.

## Materials and methods

### Source publications and data extraction

A PubMed search with the keywords “Osteopontin” and ‘splice” through June 2021 identified 36 references (Supplemental Table S1) that report measurements in patients. Variations on the search terms, including “isoform”, “splicing” or “OPN”, “spp1″ did not retrieve additional references. The data extraction was limited solely to measurements in patients. Cell lines or other model systems were excluded. Tabulated data were extracted as reported, graphed data were measured with a ruler, where raw data were available they were made use of. One author performed the data extraction. The R package dplyr' v1.0.6 (https://dplyr.tidyverse.org/) was utilized for data preparation before performing any analyses.

### Test for reporting bias

We performed effect size estimates (on the basis of a random-effects model) by generating funnel plots with the ‘metafor v2.4–0’ package in R [11]. The effect sizes were calculated with ‘escalc’, assuming the standardized mean difference

1 $${\varvec{s}}{\varvec{m}}{\varvec{d}}=\boldsymbol{ }\frac{{{\varvec{\mu}}}_{1}-{{\varvec{\mu}}}_{2}}{{\varvec{S}}-{\varvec{p}}{\varvec{o}}{\varvec{o}}{\varvec{l}}{\varvec{e}}{\varvec{d}}}$$

This was done for the comparison of “normal” (including “surrounding normal”, which provides a cancer-free reference point in the same patient) versus “cancer” as well as for tumor grade and stage.

### Categorical meta-analysis

A significance level of 95% (*p* < 0.05) was applied to all studies. The correlation between Osteopontin splice variant expression levels and the clinical variables of interest was examined with a categorical approach (using ranked values). The data ranking achieves a substantial increase in sensitivity of the analysis. Ranking accomplishes a self-normalization within each study and permits the simultaneous analysis of both the summary results (mean, median only) and various graded results. In the case of immunohistochemistry, this reduces the effects of different pathologists scoring the samples. In other assay types, such as ELISA or quantitative RT-PCR, this eliminates the need for a normal standard under the assumption that all samples within a study are compared against the same standard [1, 2].

Categorization was based on the assumption of normal distribution for all data sets. Initially, it entailed a) calculating the values of the 1/3 and 2/3 percentiles using the mean and standard deviation from the whole population (e.g. including cancer and normal), b) estimating the 1/3 and 2/3 percentiles for the whole population, using the mean and standard deviation from cancer and normal separately, c) calculating the number of subjects belonging to the various categories (low, medium, or high) for cancer and normal separately. As this approach left a non-trivial number of original reports not evaluable, we needed to relax the criteria to a) reducing the categories to high versus low, or positive versus negative, which enabled the inclusion of studies that reported only dichotomized results, b) calculating the value of the 1/2 percentile (median) using the mean and standard deviation from the whole population, c) estimating the median for the whole population, using the mean and standard deviation from each subset separately, d) calculating the number of subjects belonging to the each category separately, e) integrating the counts of the dichotomized results and numeric results in all categories. With this approach, a larger number of literature reports could be included. The resulting counts were aggregated per cancer type, so that there remained only one cancer record and one normal record for each cancer type within the data.

### Estimation of mean values and standard deviations

In the predecessor studies [1, 2], a Monte Carlo approach was used to achieve distribution-independent assessments. In the present investigations, we assumed normal distribution for all reported results. We estimated the mean values and scatters such that the integrated mean and standard deviation (std) from multiple groups were calculated as

2 $${\varvec{i}}{\varvec{n}}{\varvec{t}}{\varvec{e}}{\varvec{g}}{\varvec{r}}{\varvec{a}}{\varvec{t}}{\varvec{e}}{\varvec{d}}\ \boldsymbol{ }{\varvec{m}}{\varvec{e}}{\varvec{a}}{\varvec{n}}=\boldsymbol{ }\frac{{{\varvec{\mu}}}_{1}\boldsymbol{*}{{\varvec{n}}}_{1}+{{\varvec{\mu}}}_{2}\boldsymbol{*}{{\varvec{n}}}_{2}}{{{\varvec{n}}}_{1}+{{\varvec{n}}}_{2}}$$

3 $${\varvec{i}}{\varvec{n}}{\varvec{t}}{\varvec{e}}{\varvec{g}}{\varvec{r}}{\varvec{a}}{\varvec{t}}{\varvec{e}}{\varvec{d}}\ {\varvec{s}}{\varvec{t}}{\varvec{d}}= \sqrt{\frac{\left({{\varvec{n}}}_{1}-1\right)*{{{\varvec{\sigma}}}_{1}}^{2}+\left({{\varvec{n}}}_{2}-1\right)*{{{\varvec{\sigma}}}_{2}}^{2}}{{{\varvec{n}}}_{1}+{{\varvec{n}}}_{2}}}$$

When mean or median was reported together with range, the reported mean or median was accepted with the assumption.

4 $${\varvec{s}}{\varvec{t}}{\varvec{d}}=\frac{{\varvec{m}}{\varvec{a}}{\varvec{x}}-{\varvec{m}}{\varvec{i}}{\varvec{n}}}{4}$$

For reports of median, bottom and top quartile (Q3 or 25% and Q1 or 75%) the reported median was accepted and.

5 $${\varvec{s}}{\varvec{t}}{\varvec{d}}= \frac{{\varvec{Q}}3-{\varvec{Q}}1}{2*0.674}$$

When individual data points were reported, we used

6 $${\varvec{m}}{\varvec{e}}{\varvec{a}}{\varvec{n}}=\boldsymbol{ }\frac{\sum ({\varvec{x}}{\varvec{i}})}{{\varvec{n}}}$$

7 $${\varvec{s}}{\varvec{t}}{\varvec{d}}= \sqrt{\frac{\sum {({\varvec{x}}{\varvec{i}}-{\varvec{\mu}})}^{2}}{{\varvec{n}}-1}}$$

### Meta-analysis

The Cochran-Mantel–Haenszel χ^2^ test was used to assess the hypothesis that the ranking of a particular clinical variable within a study is linearly related to the Osteopontin variant level [12]. We utilized the Pearson χ^2^ test for independence to assess whether the Osteopontin variant ranks are independent of the clinical variable ranks. This test was carried out by constructing contingency tables using the ranks for each variable and populating each cell with the total number of patients reporting that combination of ranks. Separate tables were constructed for sets of studies with 2, 3, or more ranks to avoid structural zeros. A warning occurs, when one or more expected values are less than five. In this case, Fisher’s exact test was applied to confirm the results. For analyses that did not have sufficient entries for a Pearson χ^2^ test in the 2*2 or 2*3 table items (warnings provided in the results from R), Fisher’s exact test was applied as an alternative.

Within a study, tumor stage or grade were dichotomized as low (1–2) versus high (3–4). Stage T and stage N were likewise dichotomized, while stage M only has the values 0 or 1. The same process for integrated count calculation was applied in order to determine result categories (low, medium, or high; alternatively 1–2 = low, 3–4 = high) for stage or grade groups before implementing a Pearson χ^2^ test or a Fisher’s exact test.

### TSVdb examination

We sought to strengthen the meta-analysis from published papers with the examination of entries (including data through December 2021) in the TCGA Splicing Variants database (http://tsvdb.com/), which represents a web tool for integrating and visualizing mRNA alternative splicing, transcriptional isoform expression and clinical information from The Cancer Genome Atlas project (TCGA) RNA-Seq data [13, 14] . 33 tumor types are represented in this database.

We evaluated sample type, tumor stage, and overall survival. Spreadsheet downloads enabled the assessment of significant differences pairwise between the groups within a category via one-tailed t-test for samples assuming equal variance. To visualize sample type and stage, we selected beeswarm plots for isoform expression (normalized RSEM (RNA-Seq by Expectation–Maximization)), covering uc003hra.3 (OPN-a), uc003hrc.3 (OPN-b), uc003hrb.3 (OPN-c), uc003hrd.3 (OPN-4), and uc011cde.2 (OPN-5). The codes starting with uc0 represent UCSC gene identifications (developed at the University of California, Santa Cruz). Death from cancer was analyzed in two ways. One evaluation measured the significant differences between survivors and non-survivors over 5 years. In a second approach, the Kaplan–Meier curves set the median as the cut-off between high and low expressors. Graph components entail information on the groups including the cut-off value and sample size and the survival line for each individual included after filtering from the survival start time point.

## Results

### Literature cancer marker

**Fig. 2:**
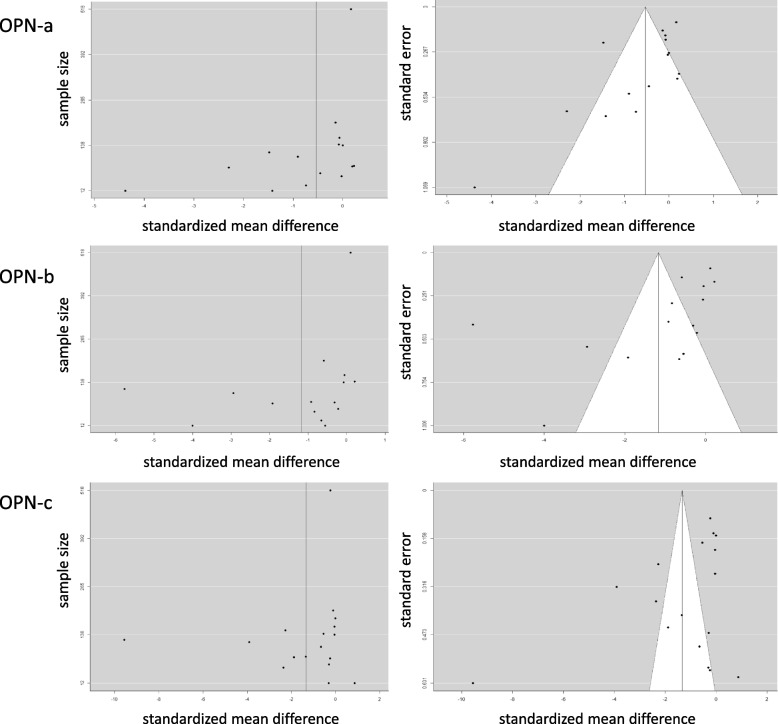
Effect size for the comparison of cancer versus normal in published literature reports. Graphs of sample size versus standardized mean difference (left panel) and canonical funnel plots (standard error versus standardized mean difference) (right panel) for Osteopontin-a (top row), -b (middle row), and -c (bottom row)

In the published literature, Osteopontin splice variants have been evaluated in 15 types of malignancies (breast cancer, colon cancer, esophageal cancer, gastric cancer, glioma, head and neck cancer, liver cancer, lung cancer, mesothelioma, ovarian cancer, cervical cancer, pancreatic cancer, prostate cancer, soft tissue sarcoma, and thyroid cancer). Assessing the comparison of cancer versus healthy, we calculated the effect sizes. For OPN-a, the estimated amount of total heterogeneity τ^2^ = 05,351 (standard error = 0.2588), total variability I^2^ = 90.34% and sampling variability H^2^ = 10.35. For OPN-b, the estimated amount of total heterogeneity τ^2^ = 2.6225 (standard error = 1.0678), total variability I^2^ = 97.66% and sampling variability H^2^ = 42.80. For OPN-c, the estimated amount of total heterogeneity τ^2^ = 5.3489 (standard error = 1.9476), total variability I^2^ = 98.99% and sampling variability H^2^ = 99.09. The effect sizes from these studies cover a range (Fig. 2), but we did not exclude any study from the analyses.

We compared cancer versus healthy (normal) with the protocol for two categories, which allowed the inclusion of dichotomized reports. For all cancers combined, the Mantel–Haenszel χ^2^ test with continuity correction had for OPN-a the test statistic χ^2^ = 11.688, *p* = 0.0006291, common odds ratio = 0.6955606 (95% confidence interval 0.5635650 to 0.8584717), for OPN-b the test statistic χ^2^ = 52.997, *p* = 3.34 × 10^–13^, common odds ratio = 0.4809881 (95% confidence interval 0.3920714 to 0.5900700), for OPN-c the test statistic χ^2^ = 188.16, p = 2.2 × 10^–16^, common odds ratio = 0.2618451 (95% confidence interval 0.2144581 to 0.3197027). For individual cancers, we also evaluated the significance of the association with Osteopontin-derived markers. For all cancers combined, each splice variant is a biomarker. OPN-a upregulation is associated with lung, liver, and pancreatic cancers. OPN-b elevation is a marker for lung and liver cancers. High levels of OPN-c are associated with breast and lung cancers (Table 1). When categorizing the data as low, medium and high, fewer original reports could be included, which limited the meta-analysis approach. With some deviations, the results are similar to the three-category analysis that included only reported values with distribution information (Supplement Table S2).

**Table 1:** Association of Osteopontin splice variants with various cancers according to published reports. Only cancers with multiple evaluable reports are listed. The left column displays the cancer type under study, the three blocks have the numbers for Osteopontin-a (OPN-a), Osteopontin-b (OPN-b), and Osteopontin-c (OPN-c). Results in bold are considered significant at the 5% probability of error level. studies = number of original reports underlying the analysis, *n* = number of patients analyzed, χ^2^ = result of the χ^2^ test, *p*-value = significance according to the χ^2^ test, “normal” indicates healthy tissue. A warning occurred when χ^2^ was below 5. In all of those cases, the *p*-value according to Fisher’s exact test corroborated the results

	**OPN-a**								**OPN-b**								**OPN-c**							
cancer	studies	n	category	low	high	χ^2^	*p*-value	Fisher p-value	studies	n	category	low	high	χ^2^	*p*-value	Fisher *p*-value	studies	n	category	low	high	χ^2^	*p*-value	Fisher p-value
breast	3	354	cancer	106	108	0.25305	0.6149	0.5871	3	354	cancer	109	105	1.3646	0.2427	0.2307	4	479	cancer	91	179	90.994	**< 2.2e-16**	** < 2.2e-16**
			normal	74	66						normal	81	59						normal	163	46			
lung	2	146	cancer	24	45	69.565	**< 2.2e-16**	**< 2.2e-16**	2	146	cancer	11	58	103.9	** < 2.2e-16**	**< 2.2e-16**	2	301	cancer	48	89	43.947	**3.37E-11**	**1.64E-11**
			normal	77	0						normal	77	0						normal	121	43			
liver	2	21	cancer	0	11	4.7256	**0.02972**	**0.01238**	2	21	cancer	0	11	4.7256	**0.02972**	**0.01238**	2	21	cancer	8	3	0.38595	0.5344	0.387
			normal	5	5						normal	5	5						normal	5	5			
pancreatic	2	127	cancer	7	40	13.261	**0.0002709**	**0.000116**	2	127	cancer	19	28	2.3665	0.124	0.09988	2	127	cancer	33	14	0.1661	0.6836	0.5655
			normal	39	41						normal	45	35						normal	52	28			
glioma	2	168	cancer	77	82	1.8784	0.1705	0.1678	2	168	cancer	75	84	2.0862	0.1486	0.0934	2	168	cancer	77	82	0.52134	0.4703	0.3259
			normal	7	2						normal	7	2						normal	6	3			
all	17	1844	cancer	503	562	18.626	**1.59E-05**	**1.41E-05**	17	1844	cancer	490	575	47.786	**4.754E-12**	**3.474E-12**	19	2274	cancer	566	743	170.35	**< 2.2e-16**	**< 2.2e-16**
			normal	448	331						normal	486	293						normal	684	281			

### TSVdb cancer marker

Analysis of the TCGA Splicing Variants database suggests significant expression changes for multiple Osteopontin splice variants in several cancers, when compared to normal tissue (Table 2). The expression of all forms is elevated in stomach adenocarcinoma. OPN-a, OPN-b, OPN-c, and OPN-5 are increased in renal papillary cell carcinoma, cholangiocarcinoma, hepatocellular carcinoma, lung adenocarcinoma, lung squamous cell carcinoma, as well as head and neck cancer. Higher abundance in OPN-a, OPN-c, and OPN-5 occurs in endometrial carcinoma, breast adenocarcinoma, colon adenocarcinoma, and glioblastoma. Further, cutaneous melanoma shows upregulation of OPN-a, OPN-b, OPN-c, and OPN-5 in the comparison of primary versus metastatic growths. OPN-a and OPN-b are lowered in renal clear cell carcinoma. Ovarian serous cystadenocarcinoma displays a reduction in OPN-a from primary to recurring tumors (only 4 recurring specimens). OPN-a, OPN-b, OPN-c, and OPN-5 are reduced in pancreatic adenocarcinoma, however, the sample numbers are very limiting (1 metastasis, 4 healthy controls).

**Table 2:** Association of Osteopontin splice variants with various cancers and their stage in TSVdb. Only the significant *p*-values (< 0.05 according to one-tailed t-test assuming equal variance) are listed. Left Panel) Comparison of cancer versus healthy (normal) tissue (h), primary tumor versus metastasis (m), or original cancer versus recurrence (r). All significant changes are high in cancer/in metastasis/in recurrence. Right panel) Significant changes in Osteopontin splice variant expression with tumor stage. Where multiple stages are significant, the highest *p*-value is listed and the smallest n. With the exception of entries in italics, all significant changes reflect increases. I/II = stage I versus stage II, I/III = stage I versus stage III, I/IV = stage I versus stage IV, II/IV = stage II versus stage IV, 6,7,8,9,10 = Gleason stages

cancer				**cancer**						**stage**		
	**n**	**OPN-a**	**OPN-b**	**OPN-c**	**OPN-4**	**OPN-5**	**n**	**OPN-a**	**OPN-b**	**OPN-c**	**OPN-4**	**OPN-5**
adrenocortical carcinoma	79											
pheochromocytoma/paraganglioma	187											
kidney chromophobe	91						41				0.039 (I/IV)	
renal clear cell carcinoma	605	11.6E-5 (h)	2.5E-5 (h)				500				15.3E-3 (I/II,I/III)	
renal papillary cell carcinoma	323	1.02E-5 (h)	2.67E-3 (h)	2.21E-6 (h)		2.77E-6 (h)	210	0.011 (I/II)		4.8E-3 (I/II)		
bladder urothelial carcinoma	427											
prostate adenocarcinoma	550						550	0.03 (6/7–6/10)	0.02 (6/7–6/10)	0.04 (6/7–6/10)		
testicular germ cell tumor	156						82	0.028 (I/II,I/III)	0.011 (I/III)	0.004 (I/III)		
ovarian serous cystadenocarcinoma	307	4.67E-2 (r)										
cervical cancers	309											
endometrial carcinoma	201	0.022 (h)		0.018 (h)		3.78E-3 (h)						
uterine carcinosarcoma	57						42				*0.02 (I/III)*	
breast adenocarcinoma	1212	6.11E-9 (h)		2.27E-10 (h)		5.45E-5 (h)	222					*0.034 (I/IV)*
esophageal carcinoma	196						163	0.028 (I/II,I/III,I/IV)	0.028 (I/II,I/III,I/IV)	0.035 (I/II,I/III,I/IV)	0.012 (I/II,I/III)	0.048 (I/II,I/III,I/IV)
stomach adenocarcinoma	450	0.019 (h)	0.039 (h)	0.034 (h)	0.029 (h)	0.043 (h)	65	0.037 (I/IV)		0.036 (I/IV)		0.022 (I/IV)
colon adenocarcinoma	328	4.53E-4 (h)		3.58E-4 (h)		4.17E-4 (h)	137	0.012 (I/III)	0.022 (I/III)	0.011 (I/III)		0.007 (I/III)
rectum adenocarcinoma	105						31	0.007 (I/IV)	0.012 (I/IV)	0.010 (I/IV)		0.023 (I/IV)
cholangiocarcinoma	45	9.90E-3 (h)	3.27E-3 (h)	22.4E-3 (h)		8.06E-3 (h)						
hepatocellular carcinoma	423	8.22E-4 (h)	6.36E-3 (h)	7.05E-3 (h)		6.69E-3 (h)	196				1.72E-4 (I/IV)	
pancreatic adenocarcinoma	183	5.15E-4 (h)	1.28E-3 (h)	3.84E-3 (h)		5.72E-2 (h)	72	0.020 (I/II)	0.016 (I/II)	0.013 (I/II)		
lung adenocarcinoma	576	1.73E-6 (h)	0.013 (h)	2.17E-6 (h)		1.02E-5 (h)						
lung squamous cell carcinoma	552	1.05E-6 (h)	0.021 (h)	3.70E-5 (h)		8.42E-3 (h)	361	*0.031 (I/II)*		0.015 (I/III)		*0.020 (I/II)*
mesothelioma	87											
thyroid carcinoma	568	0.010 (h)	0.013 (h)	0.022 (h)			382	*0.034 (I/II,I/III)*	*0.035 (I/II)*	*0.021 (I/II,I/III)*	0.008 (I/III)	0.026 (I/III)
thymoma	122						43	0.021 (I/IV)	0.025 (I/IV)	0.028 (I/IV)	0.016 (I/IV)	0.005 (I/IV)
diffuse large B-cell lymphoma	48											
acute myeloid leukemia	173											
head and neck cancer	566	0.013 (h)	0.040 (h)	9.9E-3 (h)		0.039 (h)	119	0.028 (I/II,I/IV)	0.001 (I/II)	0.025 (I/II,I/IV)		0.013 (I/II)
glioblastoma	171	37.5E-3 (h)		35.9E-3 (h)		23.4E-3 (h)						
brain lower grade glioma	530											
cutaneous melanoma	473	0.012 (m)	0.028 (m)	2.67E-3 (m)		0.01 (m)	102	0.026 (I/IV)	5.77E-3 (I/IV)		*17.9E-3 (I/II)*	
uveal melanoma	80											
sarcoma	265											

### TSVdb stage

All osteopontin splice forms change with stage in thyroid carcinoma, esophageal carcinoma, and thymoma. Stage-dependent deviations of OPN-a, OPN-b, OPN-c, and OPN-5 arise in head and neck cancer, colon adenocarcinoma, and rectum adenocarcinoma. OPN-a, OPN-b, and OPN-5 display altered expression with increasing stage in lung squamous cell carcinoma, stomach adenocarcinoma, and renal papillary cell carcinoma. Solely OPN-a, OPN-b, and OPN-c change with stage in pancreatic adenocarcinoma, prostate adenocarcinoma, and testicular germ cell tumor. OPN-4 is a marker for stage in renal clear cell carcinoma, kidney chromophobe, uterine carcinosarcoma, hepatocellular carcinoma, and – in conjunction with OPN-a and OPN-b – in cutaneous melanoma. The results are summarized in Table 2. While most changes reflect an upregulation with increasing stage, a few exceptions are marked in italics in the Table.

### Literature grade and stage

The effect sizes from the relevant studies cover a range (Supplemental Figure S1), but we did not exclude any study from the analyses. In the comparison of low versus intermediate versus high grades, for all cancers combined, the Cochran-Mantel–Haenszel test had for OPN-a M^2^ = 1.0457, df (degrees of freedom) = 2, *p* = 0.5928, for OPN-b M^2^ = 3.2225, df = 2, *p* = 0.1996 and for OPN-c M^2^ = 9.7358, df = 2, *p* = 0.00769. For individual cancers, we evaluated the significance of the association with Osteopontin-derived markers. OPN-a is associated with higher grade in glioma, breast cancer, and lung cancer. OPN-b levels increase with grade in glioma. OPN-c is a marker for grade in glioma and breast cancer (Table 3).

**Table 3:** Association of Osteopontin splice variants with cancer grade and stage in the literature. Only cancers with multiple evaluable reports for at least one splice form are listed. The left column displays the cancer type under study, the three blocks have the numbers for Osteopontin-a (OPN-a), Osteopontin-b (OPN-b), and Osteopontin-c (OPN-c). *n* = number of patients studied, χ^2^ = result of the χ^2^ test, *p*-value = result of the χ^2^ test. A warning occurred when χ^2^ was below 5. In all of those cases, the p-value according to Fisher’s exact test corroborated the results. Of note, the results from χ^2^ test or Fisher’s exact test only inform on differences in counts, not on their direction. Bottom portion) meta-analysis for tumor stage. The last column in each section indicates the analysis of overall stage, stage T (tumor growth), stage N (lymph node involvement), or stage M (metastasis, no entries) Top portion) meta-analysis for tumor grade. Results in bold are considered significant at the 5% probability of error level

	**OPN-a**									
cancer	studies	n	category	low	medium	high	χ^2^	*p*-value	Fisher *p*-value	grade
breast	1	66	Grade 1&2	12	28	6	11.45	**0.003263**	**0.002896**	
			Grade 3&4	7	4	9				
glioma	2	156	Grade 1&2	21	13	11	8.6761	**0.01306**	**0.01299**	
			Grade 3&4	28	31	52				
lung	1	35	Grade 1&2	7	7	9	8.2031	**1.66E-02**	**0.01238**	
			Grade 3&4	3	9	0				
all	5	358	Grade 1&2	76	63	70	2.5141	0.2845	0.2836	
			Grade 3&4	43	54	52				
cancer	studies	n	category	low	medium	high	χ^2^	p-value	Fisher p-value	stage
breast	1	61	low	17	16	19	15.203	**0.0005**	**0.000369**	Stage T
			high	0	9	0				
breast	1	58	low	15	14	17	11.696	**0.002886**	**0.002465**	Stage N
			high	2	10	0				
lung	1	33	low	5	5	6	0.24265	0.8857	1	Stage
			high	6	6	5				
all	2	134	low	25	37	42	9.4449	**0.008894**	**0.01332**	Stage
			high	16	7	7				

	**OPN-b**									
cancer	studies	n	category	low	medium	high	χ^2^	*p*-value	Fisher *p*-value	grade
breast	1	66	Grade 1&2	15	14	17	2.7568	0.252	0.2632	
			Grade 3&4	6	10	4				
glioma	2	156	Grade 1&2	23	12	10	17.511	**0.000158**	**0.000193**	
			Grade 3&4	22	31	58				
lung	1	35	Grade 1&2	6	11	6	3.2794	0.194	0.1988	
			Grade 3&4	5	2	5				
all	5	358	Grade 1&2	74	61	74	1.2698	0.53	0.2836	
			Grade 3&4	46	42	61				
cancer	studies	n	category	low	medium	high	χ^2^	p-value	Fisher p-value	stage
breast	1	61	low	17	17	18	0.66816	0.716	0.9003	Stage T
			high	3	4	2				
breast	1	58	low	14	16	16	0.65034	0.7224	0.7878	Stage N
			high	5	3	4				
lung	1	33	low	5	6	5	0.24265	0.8857	1	Stage
			high	6	5	6				
all	2	134	low	19	32	53	23.953	**6.29E-06**	**1.08E-05**	Stage
			high	19	6	5				

	**OPN-c**									
cancer	studies	n	category	low	medium	high	χ^2^	*p*-value	Fisher p-value	grade
breast	4	521	Grade 1&2	121	88	83	8.7687	**1.25E-02**	**1.24E-02**	
			Grade 3&4	69	71	89				
glioma	2	156	Grade 1&2	22	12	11	10.655	**0.004857**	**0.005336**	
			Grade 3&4	27	30	54				
lung	2	101	Grade 1&2	24	34	20	4.1892	0.1231	0.1228	
			Grade 3&4	8	5	10				
all	9	879	Grade 1&2	131	130	168	10.77	**0.004584**	**0.004534**	
			Grade 3&4	175	143	132				
cancer	studies	n	category	low	medium	high	χ^2^	p-value	Fisher p-value	stage
breast	3	577	low	143	148	131	2.6299	0.2685	0.2737	Stage T
			high	49	47	59				
breast	2	204	low	46	46	47	0.59581	0.7424	0.7769	Stage N
			high	7	9	6				
lung	2	110	low	12	36	10	13.767	**0.001024**	**0.000944**	Stage
			high	19	14	19				
all	3	211	low	29	37	73	29.718	**3.52E-07**	**1.03E-07**	Stage
			high	26	36	10				

In the comparison of low versus intermediate versus high stage, for all cancers combined, the Cochran-Mantel–Haenszel test had for OPN-a M^2^ = 9.57, df = 2, *p* = 0.008354 (stage M selectively M^2^ = 7.7651, df = 2, *p* = 0.0206), for OPN-b M^2^ = 23.41, df = 2, *p* = 8.252 × 10^–06^ and for OPN-c M^2^ = 19.542, df = 2, *p* = 5.707 × 10^–05^ (for OPN-c, stage M selectively M^2^ = 18.388, df = 2, *p* = 1.016 × 10^–04^). For individual cancers, we evaluated the significance of the association with Osteopontin-derived markers (Table 3). OPN-a is associated with breast cancer (stage T and stage N). OPN-c is a marker for the progression of lung cancer stage.

### TSVdb overall survival

Osteopontin has been known to be associated with risk of death in various cancers. Therefore, we analyzed the splice variants in this regard (Table 4). Alterations in OPN-a, OPN-b, OPN-c, and OPN-5 are associated with survival in lower grade glioma and cutaneous melanoma. In hepatocellular carcinoma, OPN-a, OPN-c, OPN-4, and OPN-5 are associated with survival. OPN-a, OPN-b, and OPN-c display expression changes with survival in cervical cancers. OPN-a and OPN-c are survival markers in cholangiocarcinoma and lung squamous cell carcinoma. Other outcome measures comprise colon adenocarcinoma (OPN-b and OPN-4), renal clear cell carcinoma (OPN-c and OPN-5), lung adenocarcinoma (OPN-b), renal papillary cell carcinoma (OPN-c), and adrenocortical carcinoma (OPN-4).

**Table 4:** Association of Osteopontin variants with overall survival by cancer patients in TSVdb. For each cancer in TSVdb, the Osteopontin splice variant levels were analyzed for the comparison of 5-year survival versus non-survival. The Table lists only the significant *p*-values (< 0.05) for one-tailed t-tests assuming equal variance (no entry means no significant differences were found). The second column from the left (n) indicates the number of available data sets. OPN-a, OPN-b, OPN-c, OPN-4, and OPN-5 are the splice variants captured

cancer				**survival**		
	**n**	**OPN-a**	**OPN-b**	**OPN-c**	**OPN-4**	**OPN-5**
adrenocortical carcinoma	49				0.0014	
pheochromocytoma/paraganglioma	117					
kidney chromophobe	161					
renal clear cell carcinoma	524			0.01		0.0008
renal papillary cell carcinoma	254			0.0113		
bladder urothelial carcinoma	311					
prostate adenocarcinoma	434					
testicular germ cell tumor	128					
ovarian serous cystadenocarcinoma	298					
cervical cancers	233	0.0251	0.0061	0.0152		
endometrial carcinoma	165					
uterine carcinosarcoma	21					
breast adenocarcinoma	993					
esophageal carcinoma	136					
stomach adenocarcinoma	318					
colon adenocarcinoma	268		0.0322		0.0399	
rectum adenocarcinoma	89					
cholangiocarcinoma	20	0.0063		0.0057		
hepatocellular carcinoma	314	0.0153		0.0421	0.0194	0.0124
pancreatic adenocarcinoma	148					
lung adenocarcinoma	430		0.0463			
lung squamous cell carcinoma	421	0.0338		0.0398		
mesothelioma	33					
thyroid carcinoma	465					
thymoma	113					
diffuse large B-cell lymphoma	146					
acute myeloid leukemia	0					
head and neck cancer	440					
glioblastoma	150					
brain lower grade glioma	471	2E-07	3E-07	3E-07		0.0452
cutaneous melanoma	87	0.0041	0.0112	0.0017		0.0015
uveal melanoma	166					
sarcoma	202					

Employing an alternative analytical approach, we plotted Kaplan–Meier curves (which do not dichotomize survival versus non-survival (death) in a given interval followed by correlation to splice variant expression levels as in Table 4, but do dichotomize low versus high expression levels and compare the time-lines of survival). The cut-off in all cases was the median value for each splice variant (Supplemental Figure S2). While no statistic for the survival curves is available in TSVdb, the results are consistent with those in Table 4.

## Discussion

Molecular medicine has elucidated mechanisms of oncogenesis and cancer progression that present promising points for intervention. With the increasingly abundant availability of targeted drugs for the treatment of cancer, the inhibition of these mechanisms has become actionable. Yet, to bring the potential of targeted treatment to fruition, there has been a growing need for biomarkers that can guide what medication should be given to which patient. Although the literature is filled with reports of promising cancer-relevant biomarkers, few have achieved clinical use. To be beneficial, the relevance of a biomarker needs to be backed by sufficient data, and its presence or absence must influence patient care. Osteopontin splice variants play important roles in cancer progression. In view of the past difficulties in targeting pan-Osteopontin, the alternatively spliced forms have become the focus of some research. Lead compounds are available for inhibition. It is important, however, that these variants be backed by a rigorous pool of data that identifies those patients, whose cancer progresses under the effect of specific Osteopontin forms. Our meta-analysis takes stock of the current knowledge base in this area of cancer.

Osteopontin has been known to be an indicator for the progression and metastasis of various cancers. However, its role as a Th1 inducer cytokine [3] and its variable posttranslational decorations have limited its potential utility as a cancer progression biomarker. The occurrence of splicing selectively in transformed cells conveys improved diagnostic promise to the spliced variant forms, because there is no baseline noise. As gene transcription and RNA splicing are not functionally linked, Osteopontin variant abundance may arise in a spectrum of permutations. Furthermore, while the not alternatively spliced form, Osteopontin-a, is always present when the gene is transcribed, it is highly context-dependent which of the splice variants are produced at all. The combination of elevated versus absent splice variants may differentiate among cancer types.

Here, we utilize the body of knowledge accrued on Osteopontin splice variants in cancer, derived from 36 PubMed-indexed journal articles, which report on 5886 patients across 15 tumor types, as well as from the database TSVdb, where 10,446 patient data across 33 cancer types are listed (Supplemental Table S3). The two sources are in agreement on the elevation of OPN-a, OPN-b, and OPN-c in lung cancer and the elevation of OPN-c in breast cancer as compared to healthy tissue. For some malignancies, original reports have been rather consistent in associating certain Osteopontin splice variants. This pertains particularly to OPN-a and OPN-b (but not OPN-c) in lung cancer. Unexpectedly, this meta-analysis has yielded results that are mostly but not entirely affirmative of prior notions, and are not entirely consistent between the two data sources.

- Despite a non-trivial size of the patient pool, in some cases the power may not have sufficed to show significance in the present analysis (insufficient number of studies), where associations actually do exist. Possibly for this reason, the categorical meta-analysis finds fewer variants than TSVdb to be cancer markers of thyroid (none or OPN-a versus OPN-a, OPN-b, and OPN-c) as well as liver (OPN-a and OPN-b versus OPN-a, OPN-b, and OPN-c).
- Conversely, where significant associations have been found in the present study, although they were not implied in prior reports, noisy data could be a factor. For the -omics scale data collections that underlie the splice variant database, false positive results are a possibility. Alternatively, the patient numbers from the original reports varied widely, and some may have been compromised by limited or skewed patient access. In the cases of persisting discrepancies, further investigation is required to clarify the Osteopontin splice variant utilization by those malignancies, so that their diagnostic, prognostic and possibly predictive potential can be brought to fruition.

Individual research reports apply their own methodology, based on patient and reagent availability, preferred techniques of analysis and other factors. Some results cannot be captured in meta-analytic evaluation. We did not include in this study measurements of OPN-a/b [15–17] or the comparison to pan-Osteopontin [18–26]. OPN-4 and OPN-5 were reported in too few papers [27, 28] to be amenable to categorical meta-analysis (they are however included in the TSVdb evaluations).

Progression and recurrence have been studied in breast cancer [17, 18, 29–32], gastric cancer [33], liver cancer [20, 28], mesothelioma [34], pancreatic cancer [35], and soft tissue sarcoma [26, 36]. Osteopontin variants have been associated with outcome (survival or recurrence) in breast cancer [15–18, 30–32], gastric cancer [33], glioma [19], lung cancer [37], mesothelioma [34], pancreatic cancer [35], and soft tissue sarcoma [36]. Several reports have looked at Osteopontin variants in association with other factors, such as age [18, 30, 38–40], gender [26, 36, 38–40], lifestyle [39], underlying conditions [24, 25, 35, 41], or additional marker molecules [26]. They are not covered in this meta-analysis, mostly due to lack of power. For select cancers, the TSVdb database contains information on race, gender, lifestyle, and other markers, which we have not included.

Not captured in the present meta-analysis are reports that studied Osteopontin splice variants only in cancer cell lines [38, 42–53]. While important functional information could be gleaned from those reports, the present investigation focuses solely on the clinical results.

## Conclusions

There are cases of persisting discrepancies, which require further investigation to clarify the Osteopontin splice variant utilization, so that their diagnostic, prognostic and potentially predictive potential can be brought to fruition. 

## Supplementary Information

**Additional file 1:**

## Data Availability

The datasets generated during and/or analysed during the current study are available in the published literature and databases (TSVdb).

## Abbreviations

mean: Mean value
n: Number of patients analyzed
OPN: Osteopontin
RSEM: RNA-Seq by Expectation–Maximization
smd: Standardized mean difference
SPP1 or spp1: Secreted phosphoprotein 1
std: Standard deviation
Th1: Type 1 helper T-cell
