# Supplementary material for: Meta-analysis of Osteopontin splice variants in cancer
Source: BMC Cancer. 2023 Apr 24;23:373. doi: 10.1186/s12885-023-10854-x (PMC10124019; doi:10.1186/s12885-023-10854-x)
Supplement: Supplementary file 1 — Additional file 1: [file 12885_2023_10854_MOESM1_ESM.pdf]

Supplement

Table S1: Source references for the meta-analysis of Osteopontin splice variants as cancer markers. A PubMed search with the keywords “Osteopontin” and ‘splice” through 2020 identified 36 references that contain patient data. These source publications are listed in chronologic order.

Table S2: Categorical meta-analysis of cancer versus normal. The left column displays the cancer type under study, the three blocks have the numbers for Osteopontin-a (OPNa), Osteopontin-b (OPNb), and Osteopontin-c (OPNc). Results in bold are considered significant at the 5% probability of error level. studies = number of original reports underlying the analysis (the availability of multiple studies is indicated in bold italic), n = number of patients analyzed, χ^2 = result of the χ^2 test, p-value = result of the χ^2 test. A warning occurred when χ^2 was below 5. In all of those cases, the p-value according to Fisher's exact test corroborated the results. Categorization was based on the assumption of normal distribution for all data sets. It entailed a) calculating the values of the 1/3 and 2/3 percentiles using the mean and standard deviation from the whole population (e.g. including cancer and normal), b) estimating the 1/3 and 2/3 percentiles for the whole population, using the mean and standard deviation from cancer and normal separately, c) calculating the number of subjects belonging to the various categories (low, medium, or high) for cancer and normal separately. The resulting counts were aggregated per cancer type, so that there remained only one cancer record and one normal record for each cancer type within the data. The Cochran-Mantel-Haenszel test had for OPN-a the test statistic M^2 = 30.539, degrees of freedom df = 2, probability of error p = 2.337 x 10^{-07}, for OPN-b M^2 = 48.314, df = 2, p = 3.226 x 10^{-11} and for OPN-c M^2 = 283.58, df = 2, p = 2.2 x 10^{-16}.

Table S3: Summary of the results. A) Categorical meta-analysis. In the stage column, - = stage, T = stage T, N = stage N, there are no entries for stage M. **B)** TSVdb. In the cancer column, h = cancer versus normal (healthy), r = cancer versus recurrent, m = primary versus metastatic. In the stage column, I/II = stage I versus stage II, I/III = stage I versus stage III, I/IV = stage I versus stage IV, II/IV = stage II versus stage IV, 6,7,8,9,10 = Gleason stages. In the survival column, 0/1 = death versus 5-year survival.

Figure S1: Effect size for the comparison of increasing grades. For Osteopontin-a (**left panel**), -b (**middle panel**), and -c (**right panel**), graphs of sample size versus standardized mean difference (left) and canonical funnel plots (standard error versus standardized mean difference) (right) are displayed for the comparisons between various grade levels.

Figure S2: TSVdb analysis. Evaluative graphs for the 33 cancer types in the TCGA Splicing Variants database. **S2.1 through S2.33)** Each column reflects the analysis of one Osteopontin splice variant. The two top rows show beeswarm plots for sample type and pathological stage. In the bottom row, the Kaplan-Meier survival curves have set the median expression level of the pertinent Osteopontin splice variant as the cut-off between high and low expressors. Graph components entail information on the groups, including sample size (above) and the survival line for each individual included after filtering from the survival start time point (below). Each page represents the evaluation of an individual cancer type (indicated on the top left).

authors	title	journal	year	volume	pages	cancer	analyte	technique
Mirza M, Shaughnessy E, Hurley JK, Vanpatten KA, Pestano GA, He B, Weber GF	Osteopontin-c is a selective marker of breast cancer	Int. J. Cancer	2008	122	889-897	breast cancer	tumor RNA, tissue	RT-PCR, IHC
Patani N, Jiang W,Mokbel K	Osteopontin C mRNA expression is associated with a poor clinical outcome in human breast cancer	Int. J. Cancer	2008	122	2646	breast cancer	tumor RNA	RT-PCR
Patani N, Jouhra F, Jiang W, Mokbel K	Osteopontin Expression Profiles Predict Pathological and Clinical Outcome in Breast Cancer	Anticancer Res	2008	28	4105-4110	breast cancer	tumor RNA	RT-PCR
Pang H, Lu H, Song H, Meng Q, Zhao Y, Liu N, Lan F, Liu Y, Yan S, Dong X, Cai L	Prognostic values of osteopontin-c, E-cadherin and β-catenin in breast cancer	Cancer Epidemiol	2013	37	985-992	breast cancer	tumor tissue	IHC
Ortiz-Martínez F, Perez-Balaguer A, Ciprián D, Andrés L, Ponce J, Adrover E, Sánchez-Payá J, Aranda FI, Lerma E, Peiró G	Association of increased osteopontin and splice variant-c mRNA expression with HER2 and triple-negative/basal-like breast carcinomas subtypes and recurrence	Hum Pathol	2014	45	504-512	breast cancer	tumor RNA, tissue	RT-PCR, IHC
Zduniak K, Ziolkowski P, Ahlin C, Agrawal A, Agrawal S, Blomqvist C, Fjallskog ML, Weber GF	Nuclear osteopontin-c is a prognostic breast cancer marker	Brit J Cancer	2015	112	729-738	breast cancer	tumor tissue	IHC
Zduniak K, Agrawal A, Agrawal S, Hossain MM, Ziolkowski P, Weber GF	Osteopontin splice variants are differential predictors of breast cancer treatment responses	BMC Cancer	2016	16	441	breast cancer	tumor tissue	IHC
Walaszek K, Lower EE, Ziolkowski P, Weber GF	Breast cancer risk in premalignant lesions: osteopontin splice variants indicate prognosis	Brit J Cancer	2018	119	1259-1266	breast cancer	tumor tissue	IHC
Chang S, Huang J, Niu H, Wang J, Si Y, Bai Z, Cheng S, Ding W	Epigenetic regulation of osteopontin splicing isoform c defines its role as a microenvironmental factor to promote the survival of colon cancer cells from 5-FU treatment	Cancer Cell Int	2020	20	452	colon cancer	tumor RNA	RT-PCR
Zhang M-X, Xu Y-J, Zhu M-C, Yan F	Overexpressed Ostepontin-c as a Potential Biomarker for Esophageal Squamous Cell Carcinoma	Asian Pac J Cancer Prev	2013	14	7315-7319	esophageal cancer	tumor tissue, blood	IHC, RT-PCR
Lin J, Myers AL, Wang Z, Nancarrow DJ, Ferrer-Torres D, Handlogten A, Leverenz K, Bao J, Thomas DG, Wang TD, Orringer MB, Reddy RM, Chang AC, Beer DG, Lin L	Osteopontin (OPN/SPP1) isoforms collectively enhance tumor cell invasion and dissemination in esophageal adenocarcinoma	Oncotarget	2015	6	22239-22257	esophageal cancer	tumor tissue	IHC
Tang X, Li J, Yu B, Su L, Yu Y, Yan M, Liu B, Zhu Z	Osteopontin splice variants differentially exert clinicopathological features and biological functions in gastric cancer	Int J Biol Sci	2013	9	55-66	gastric cancer	tumor RNA	RT-PCR
Sun X, Wang L, Hou W, Li Y, Liu L, Zuo W, Yu J	[Expression of osteopontin splice variant and its clinical significance in gastric cancer]	Zhonghua Zhong Liu Za Zhi	2015	37	427-430	gastric cancer	tumor RNA	RT-PCR
Sreekanthreddy P, Srinivasan H, Kumar DM, Nijaguna MB, Sridevi S, Vrinda M, Arivazhagan A, Balasubramaniam A, Hegde AS, Chandramouli BA, Santosh V, Rao MR, Kondaiah P, Somasundaram K	Identification of potential serum biomarkers of glioblastoma: serum osteopontin levels correlate with poor prognosis	Cancer Epidemiol Biomarkers Prev	2010	19	1409-1422	glioblastoma	tumor RNA, tissue	RT-PCR, IHC
Kijewska M, Kocyk M, Kloss M, Stepniak K, Korwek Z, Polakowska R, Dabrowski M, Gieryng A, Wojtas B, Ciechomska IA, Kaminska B	The embryonic type of SPP1 transcriptional regulation is re-activated in glioblastoma	Oncotarget	2017	8	16340-16355	glioblastoma	tumor RNA	RT-PCR
Saitoh Y, Kuratsu J, Takeshima H, Yamamoto S, Ushio Y	Expression of osteopontin in human glioma. Its correlation with the malignancy	Lab Invest	1995	72	55-63	glioma	tumor RNA	Northern blot
Yan W, Qian C, Zhao P, Zhang J, Shi L, Qian J, Liu N, Fu Z, Kang C, Pu P, You Y	Expression pattern of osteopontin splice variants and its functions on cell apoptosis and invasion in glioma cells	Neuro Oncol	2010	12	765-775	glioma	tumor RNA	RT-PCR
Güttler A, Giebler M, Cuno P, Wichmann H, Keßler J, Ostheimer C, Söling A, Strauss C, Illert J, Kappler M, Vordermark D, Bache M	Osteopontin and splice variant expression level in human malignant glioma: radiobiologic effects and prognosis after radiotherapy	Radiother Oncol	2013	108	535-540	glioma	tumor RNA	RT-PCR
Courter D, Cao H, Kwok S, Kong C, Banh A, Kuo P, Bouley DM, Vice C, Brustugun OT, Denko NC, Koong AC, Giaccia A, Le QT	The RGD domain of human osteopontin promotes tumor growth and metastasis through activation of survival pathways	PLoS One	2010	5	e9633	head and neck cancer	tumor RNA	RT-PCR
Takafuji V, Forgues M, Unsworth E, Goldsmith P, Wang XW	An osteopontin fragment is essential for tumor cell invasion in hepatocellular carcinoma	Oncogene	2007	26	6361-6371	liver cancer	tumor RNA	RT-PCR
Chae S, Jun HO, Lee EG, Yang SJ, Lee DC, Jung JK, Park KC, Yeom YI, Kim KW	Osteopontin splice variants differentially modulate the migratory activity of hepatocellular carcinoma cell lines	Int J Oncol	2009	35	1409-1416	liver cancer	tumor RNA	RT-PCR
Phillips RJ, Helbig KJ, Van der Hoek KH, Seth D, Beard MR	Osteopontin increases hepatocellular carcinoma cell growth in a CD44 dependant manner	World J Gastroenterol	2012	18	3389-3399	liver cancer	tumor RNA	RT-PCR
Goparaju CM, Pass HI, Blasberg JD, Hirsch N, Donington JS	Functional heterogeneity of osteopontin isoforms in non-small cell lung cancer	J Thorac Oncol	2010	5	1516-1523	lung cancer	tumor RNA	RT-PCR
Zhao B, Sun T, Meng F, Qu A, Li C, Shen H, Jin Y, Li W	Osteopontin as a potential biomarker of proliferation and invasiveness for lung cancer	J Cancer Res Clin Oncol	2011	137	1061-1070	lung cancer	tumor tissue	IHC
Wu J, Pungaliya P, Kraynov E, Bates B	Identification and quantification of osteopontin splice variants in the plasma of lung cancer patients using immunoaffinity capture and targeted mass spectrometry	Biomarkers	2012	17	125-133	lung cancer		
Huang J, Chang S, Lu Y, Wang J, Si Y, Zhang L, Cheng S, Jiang WG	Enhanced osteopontin splicing regulated by RUNX2 is HDAC-dependent and induces invasive phenotypes in NSCLC cells	Cancer Cell Int	2019	19	306	lung cancer	tumor RNA	RT-PCR
Ivanov SV, Ivanova AV, Goparaju CM, Chen Y, Beck A, Pass HI	Tumorigenic properties of alternative osteopontin isoforms in mesothelioma	Biochem Biophys Res Commun	2009	382	514-518	mesothelioma	tumor RNA	RT-PCR
Tilli TM, Franco VF, Robbs BK, Wanderley JL, da Silva FR, de Mello KD, Viola JP, Weber GF, Gimba ER	Osteopontin-c splicing isoform contributes to ovarian cancer progression	Mol Cancer Res	2011	9	280-293	ovarian cancer	tumor RNA	RT-PCR
Sullivan J, Blair L, Alnajar A, Aziz T, Ng CY, Chipitsyna G, Gong Q, Witkiewicz A, Weber GF, Denhardt DT, Yeo CJ, Arafat HA	Expression of a prometastatic splice variant of osteopontin, OPNC, in human pancreatic ductal adenocarcinoma	Surgery	2009	146	232-240	pancreatic cancer	tumor RNA	RT-PCR
Sullivan J, Blair L, Alnajar A, Aziz T, Chipitsyna G, Gong Q, Yeo CJ, Arafat HA	Expression and regulation of nicotine receptor and osteopontin isoforms in human pancreatic ductal adenocarcinoma	Histol Histopathol	2011	26	893-904	pancreatic cancer	tumor RNA	RT-PCR
Siddiqui AA, Jones E, Andrade D, Shah A, Kowalski TE, Loren DE, Chipitsyna G, Arafat HA	Osteopontin splice variant as a potential marker for metastatic disease in pancreatic adenocarcinoma	J Gastroenterol Hepatol	2014	29	1321-1327	pancreatic cancer	tumor RNA	RT-PCR
Sarosiek K, Jones E, Chipitsyna G, Al-Zoubi M, Kang C, Saxena S, Gandhi AV, Sendiky J, Yeo CJ, Arafat HA	Osteopontin (OPN) isoforms, diabetes, obesity, and cancer; what is one got to do with the other? A new role for OPN	J Gastrointest Surg	2015	19	639-650	pancreatic cancer	serum	RT-PCR
Tilli TM, Thuler LC, Matos AR, Coutinho-Camillo CM, Soares FA, da Silva EA, Neves AF, Goulart LR, Gimba ER	Expression analysis of osteopontin mRNA splice variants in prostate cancer and benign prostatic hyperplasia	Exp Mol Pathol	2012	92	13-19	prostate cancer	tumor RNA, tissue	RT-PCR, IHC
Hahnel A, Wichmann H, Greither T, Kappler M, Würl P, Kotzsch M, Taubert H, Vordermark D, Bache M	Prognostic impact of mRNA levels of osteopontin splice variants in soft tissue sarcoma patients	BMC Cancer	2012	12	131	soft tissue sarcoma	tumor RNA	RT-PCR
Ferreira LB, Eloy C, Pestana A, Lyra J, Moura M, Prazeres H, Tavares C, Sobrinho-Simões M, Gimba E, Soares P	Osteopontin expression is correlated with differentiation and good prognosis in medullary thyroid carcinoma	Eur J Endocrinol	2016	174	551-561	thyroid cancer	tumor RNA, tissue	RT-PCR, IHC
Hartung F, Weber GF	RNA blood levels of osteopontin splice variantsare cancer markers	SpringerPlus	2013	2	110	various cancers	blood	RT-PCR

Table S2

cancer	OPN-a						OPN-b						OPN-c					
	studies	n	χ^2	p-value	warning	Fisher p-value	studies	n	χ^2	p-value	warning	Fisher p-value	studies	n	χ^2	p-value	warning	Fisher p-value
breast	***3***	354	9.3664	**0.00925**	N	**0.00962**	***3***	354	2.5641	0.2775	N	0.2845	***3***	354	44.585	**2.08E-10**	N	**3.74E-11**
glioma	***2***	168	2.8691	0.2382	Y	0.2219	***2***	168	5.1956	0.07444	Y	0.1103	***2***	168	2.7208	0.2566	Y	0.2739
lung	***2***	146	62.382	**2.84E-14**	N	**< 2.2e-16**	1	27	4.9091	0.0859	Y	0.09709	***2***	301	128.04	**< 2.2e-16**	N	**< 2.2e-16**

Table S3A

cancer	cancer OPN-a	cancer OPN-b	cancer OPN-c	stage OPN-a	stage OPN-b	stage OPN-c	grade OPN-a	grade OPN-b	grade OPN-c
**lung**	x	x	x			x (-)	x		
**liver**	x	x							
**glioma**							x	x	x
**breast**			x	x (T,N)			x		x
**colon**									
**esophageal**									
**pancreatic**	x								
**gastric**									
**prostate**									
**ovarian / cervical**									
**head and neck cancer**									
**mesothelioma**									
**thyroid**									
**soft tissue sarcoma**									

cancer	cancer					stage					survival				
	OPN-a	OPN-b	OPN-c	OPN-4	OPN-5	OPN-a	OPN-b	OPN-c	OPN-4	OPN-5	OPN-a	OPN-b	OPN-c	OPN-4	OPN-5
**stomach adenocarcinoma**	x (h)	x (h)	x (h)	x (h)	x (h)	x (I/IV)		x (I/IV)		x (I/IV)					
**head and neck cancer**	x (h)	x (h)	x (h)		x (h)	x (I/II,I/IV)	x (I/II)	x (I/II,I/IV)		x (I/II)					
**pancreatic adenocarcinoma**	x (h)	x (h)	x (h)		x (h)	x (I/II)	x (I/II)	x (I/II)							
**cutaneous melanoma**	x (m)	x (m)	x (m)		x (m)	x (I/IV)	x (I/IV)		x (I/II)		x (0/1)	x (0/1)	x (0/1)		x (0/1)
**lung squamous cell carcinoma**	x (h)	x (h)	x (h)		x (h)	x (I/II)		x (I/III)		x (I/II)	x (0/1)		x (0/1)		
**renal papillary cell carcinoma**	x (h)	x (h)	x (h)		x (h)	x (I/II)		x (I/II)					x (0/1)		
**hepatocellular carcinoma**	x (h)	x (h)	x (h)		x (h)				x (I/IV)		x (0/1)		x (0/1)	x (0/1)	x (0/1)
**cholangiocarcinoma**	x (h)	x (h)	x (h)		x (h)						x (0/1)		x (0/1)		
**lung adenocarcinoma**	x (h)	x (h)	x (h)		x (h)							x (0/1)			
**thyroid carcinoma**	x (h)	x (h)	x (h)			x (I/II,I/III)	x (I/II)	x (I/II,I/III)	x (I/III)	x (I/III)					
**renal clear cell carcinoma**	x (h)	x (h)							x (I/II,I/III)				x (0/1)		x (0/1)
**endometrial carcinoma**	x (h)		x (h)		x (h)										
**breast adenocarcinoma**	x (h)		x (h)		x (h)					x (I/IV)					
**colon adenocarcinoma**	x (h)		x (h)		x (h)	x (I/III)	x (I/III)	x (I/III)		x (I/III)		x (0/1)		x (0/1)	
**glioblastoma**	x (h)		x (h)		x (h)										
**ovarian serous cystadenocarcinoma**	x (r)														
**esophageal carcinoma**						x (I/II,I/III,I/IV)	x (I/II,I/III,I/IV)	x (I/II,I/III,I/IV)	x (I/II,I/III)	x (I/II,I/III,I/IV)					
**thymoma**						x (I/IV)	x (I/IV)	x (I/IV)	x (I/IV)	x (I/IV)					
**rectum adenocarcinoma**						x (I/IV)	x (I/IV)	x (I/IV)		x (I/IV)					
**prostate adenocarcinoma**						x (6/7-6/10)	x (6/7-6/10)	x (6/7-6/10)							
**testicular germ cell tumor**						x (I/II,I/III)	x (I/III)	x (I/III)							
**kidney chromophobe**									x (I/IV)						
**uterine carcinosarcoma**									x (I/III)						
**adrenocortical carcinoma**														x (0/1)	
**cervical cancers**											x (0/1)	x (0/1)	x (0/1)		
**brain lower grade glioma**											x (0/1)	x (0/1)	x (0/1)		x (0/1)

Figure S1

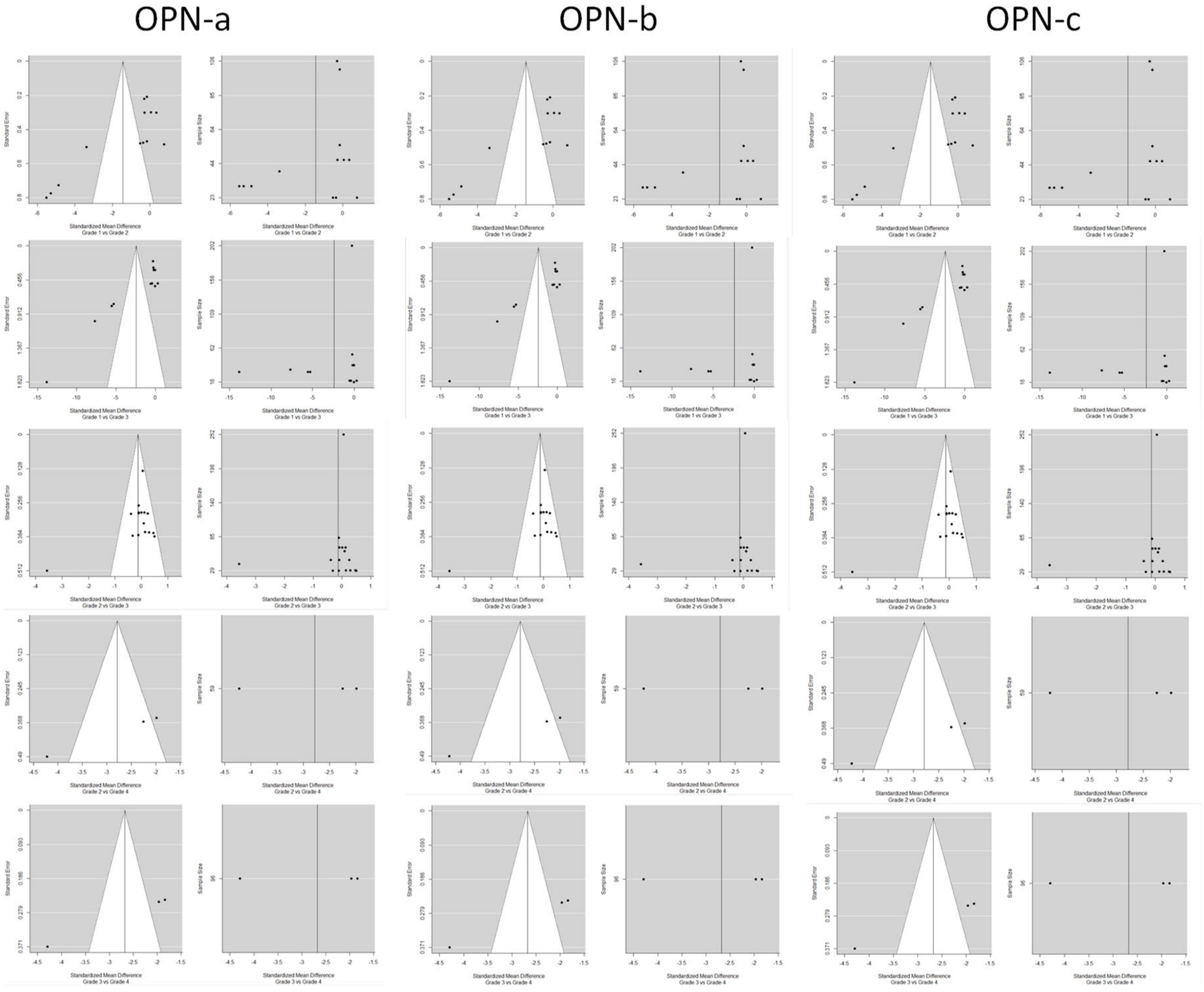

S2.1 adrenocortical carcinoma

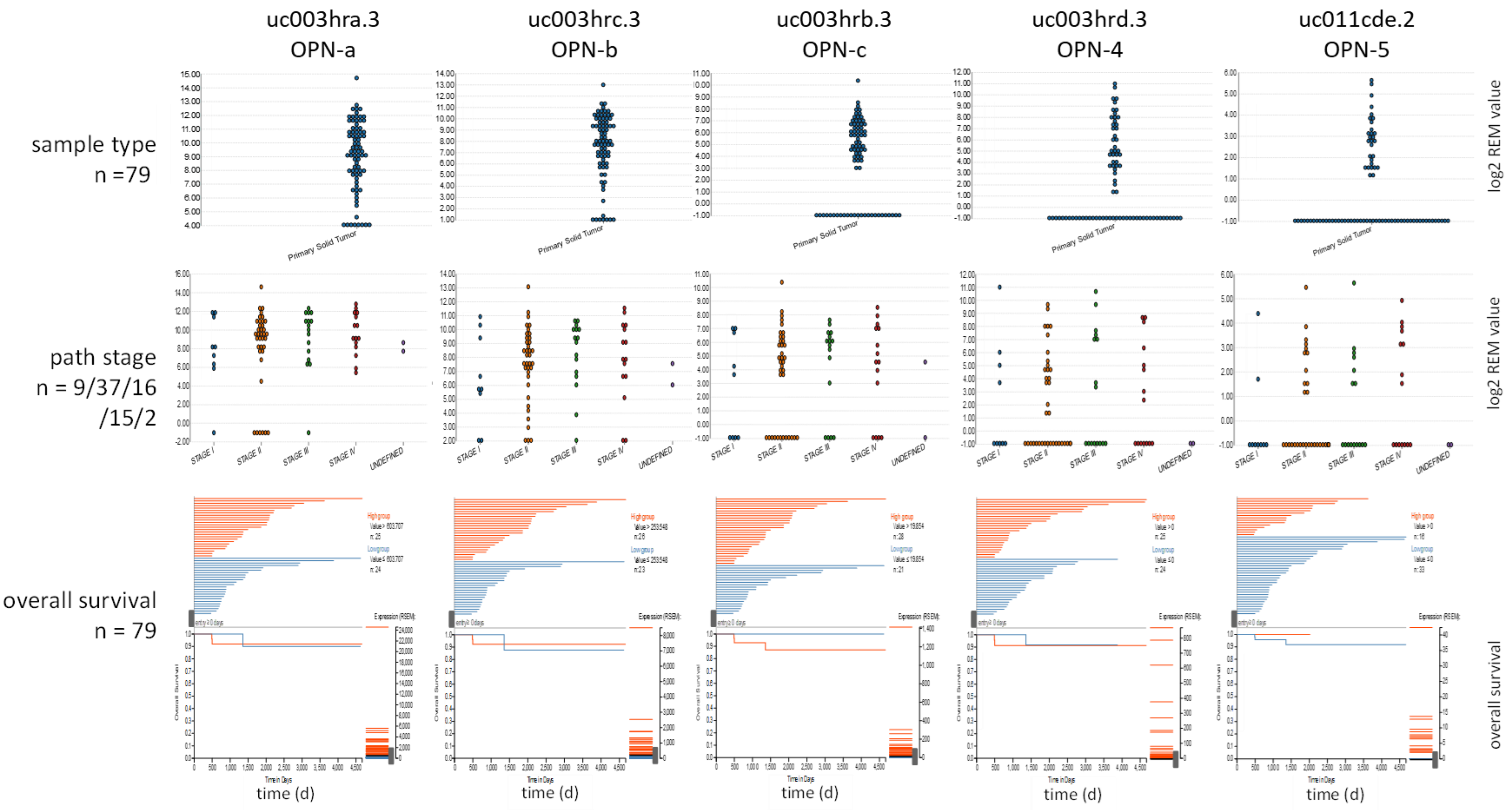

S2.2 pheochromocytoma and paraganglioma

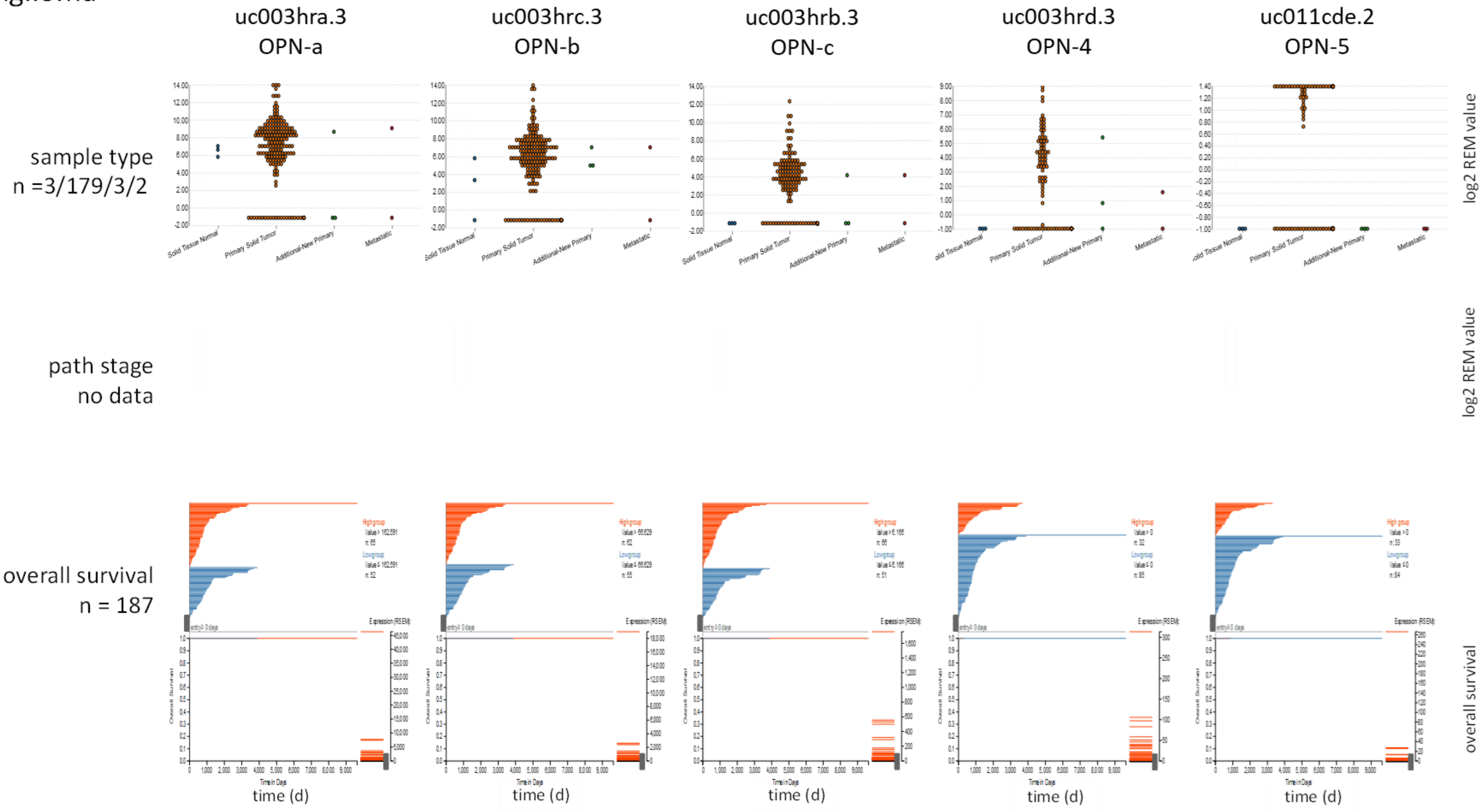

S2.3 kidney chromophobe

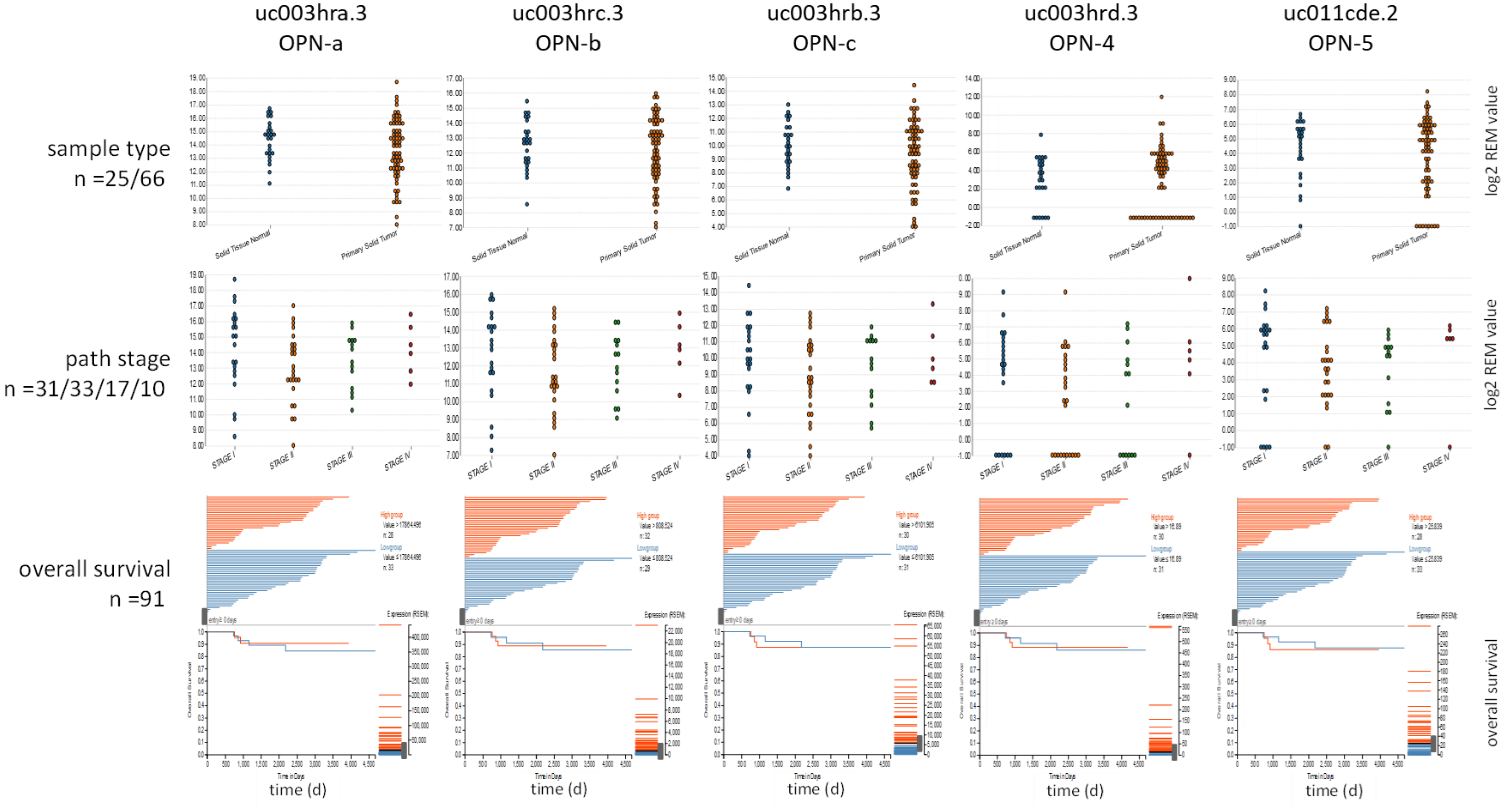

S2.4 renal clear cell carcinoma

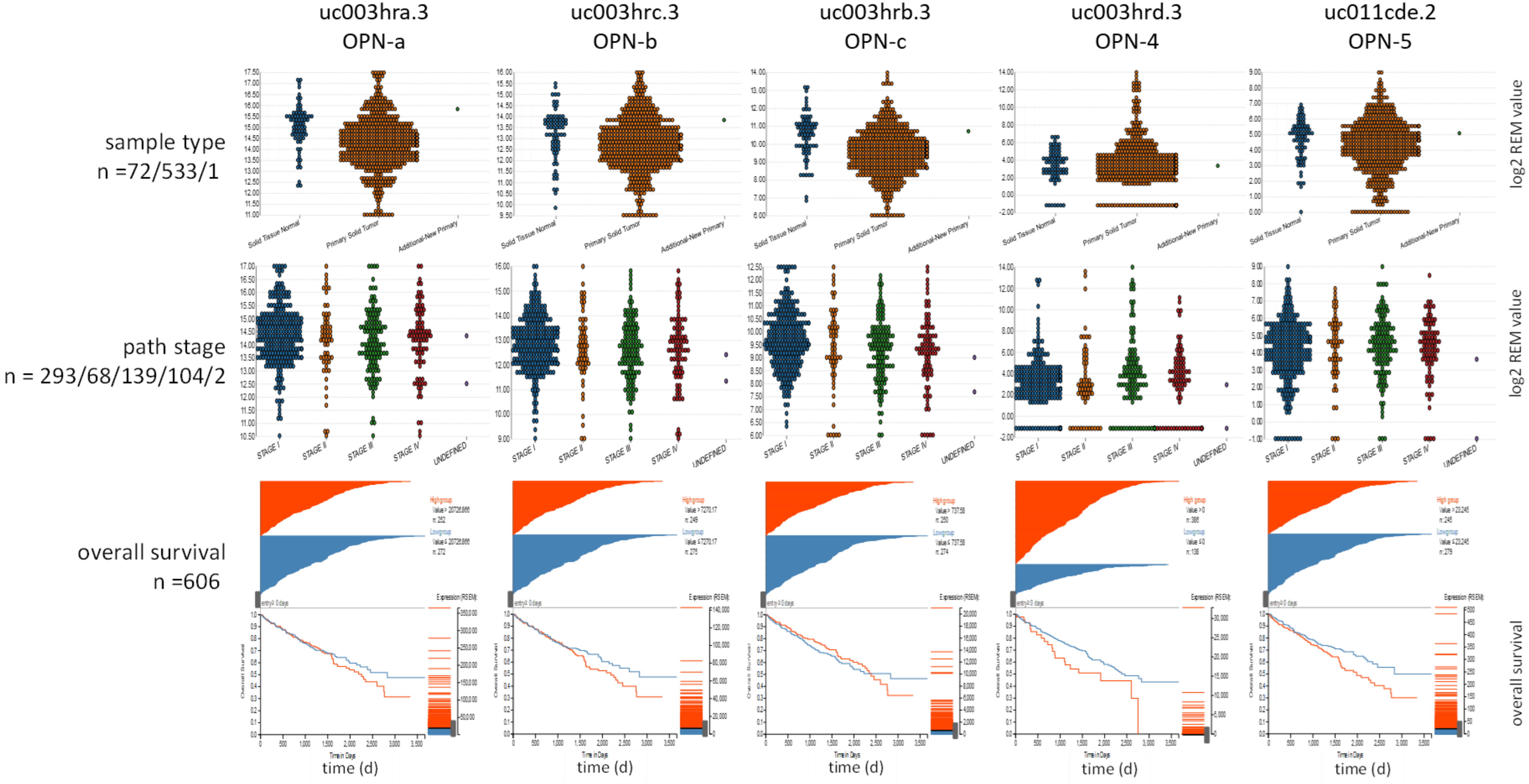

S2.5 renal papillary cell carcinoma

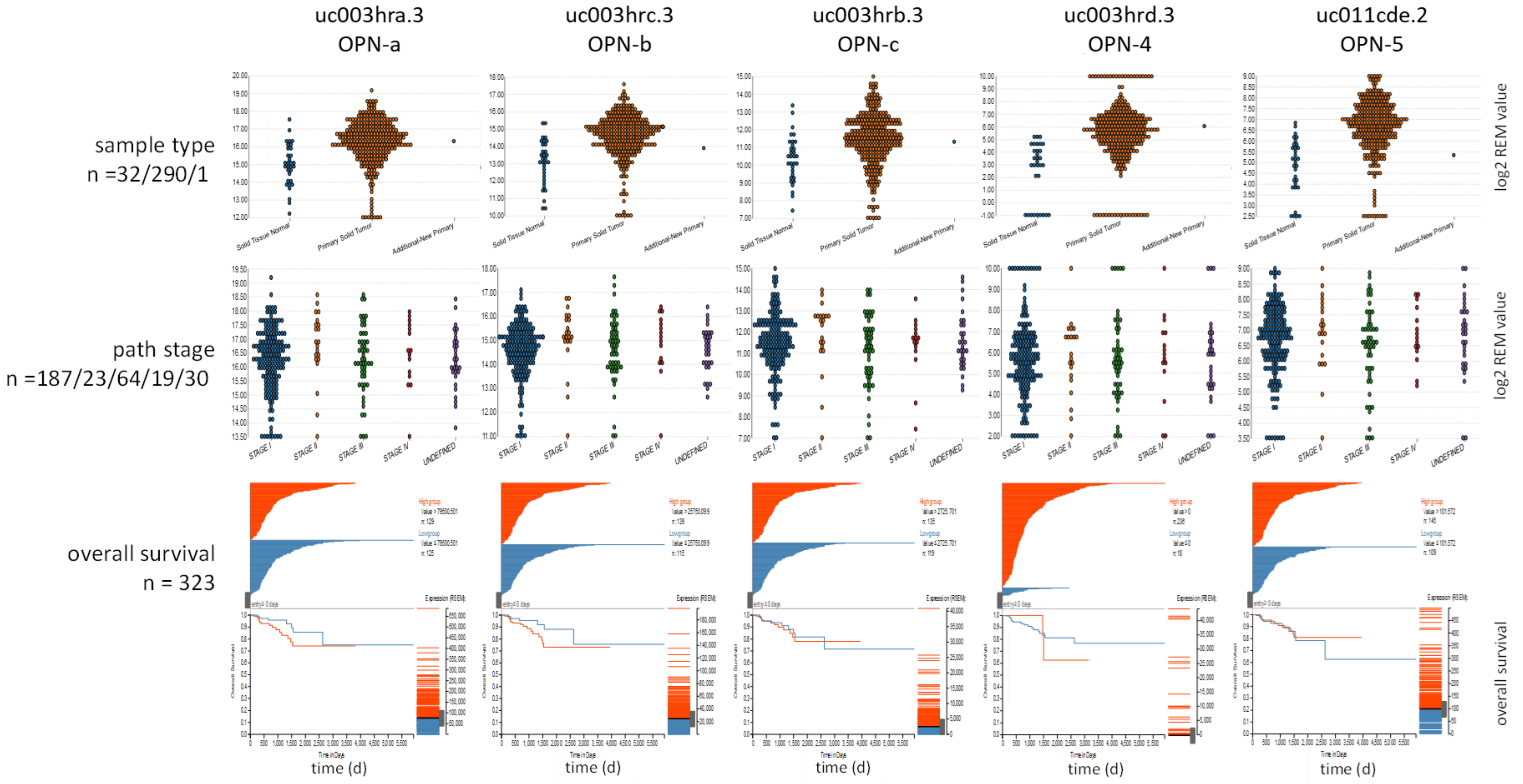

S2.6 bladder urothelial carcinoma

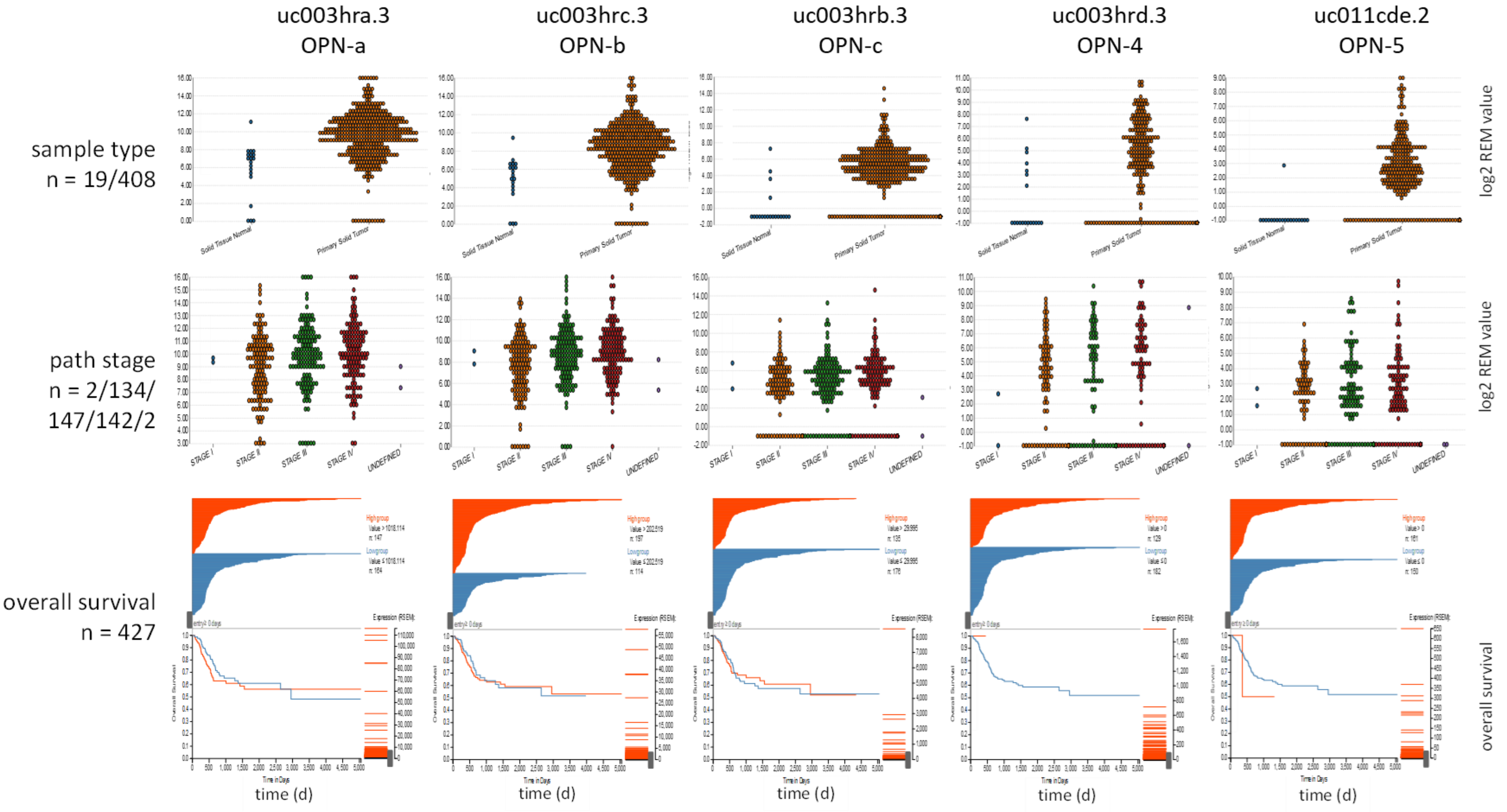

S2.7 prostate adenocarcinoma

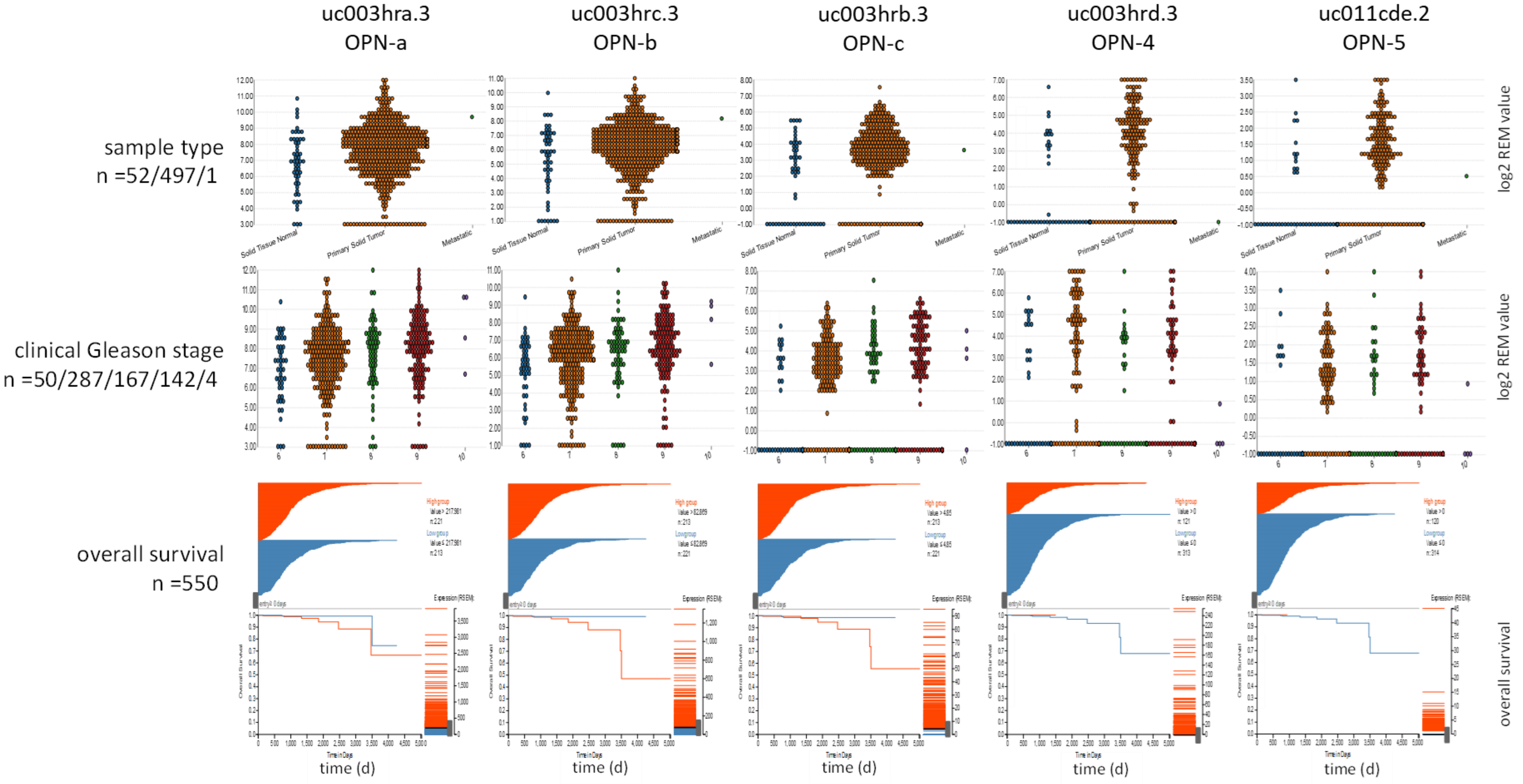

S2.8 testicular germ cell tumors

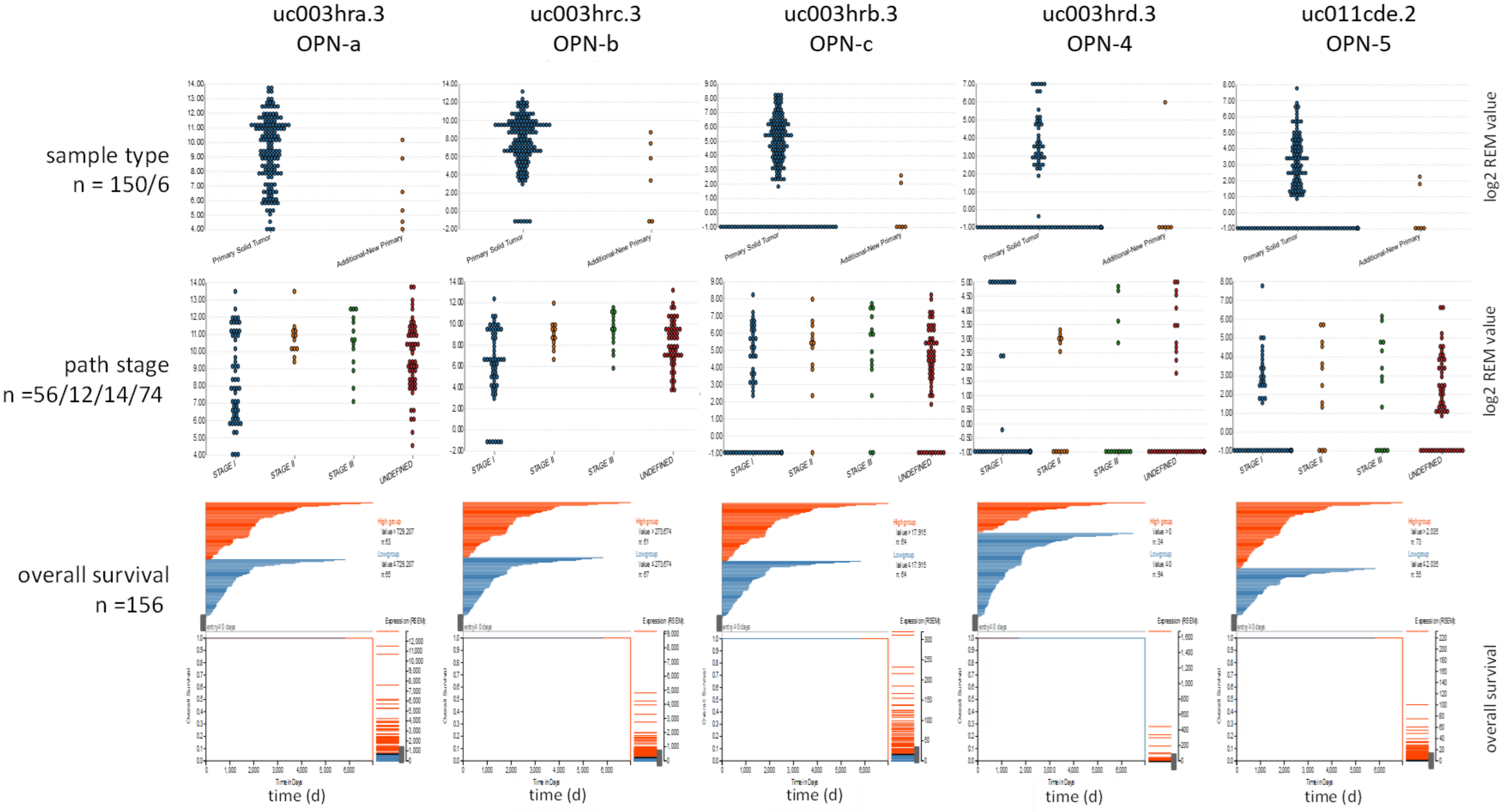

S2.9 ovarian serous cystadenocarcinoma

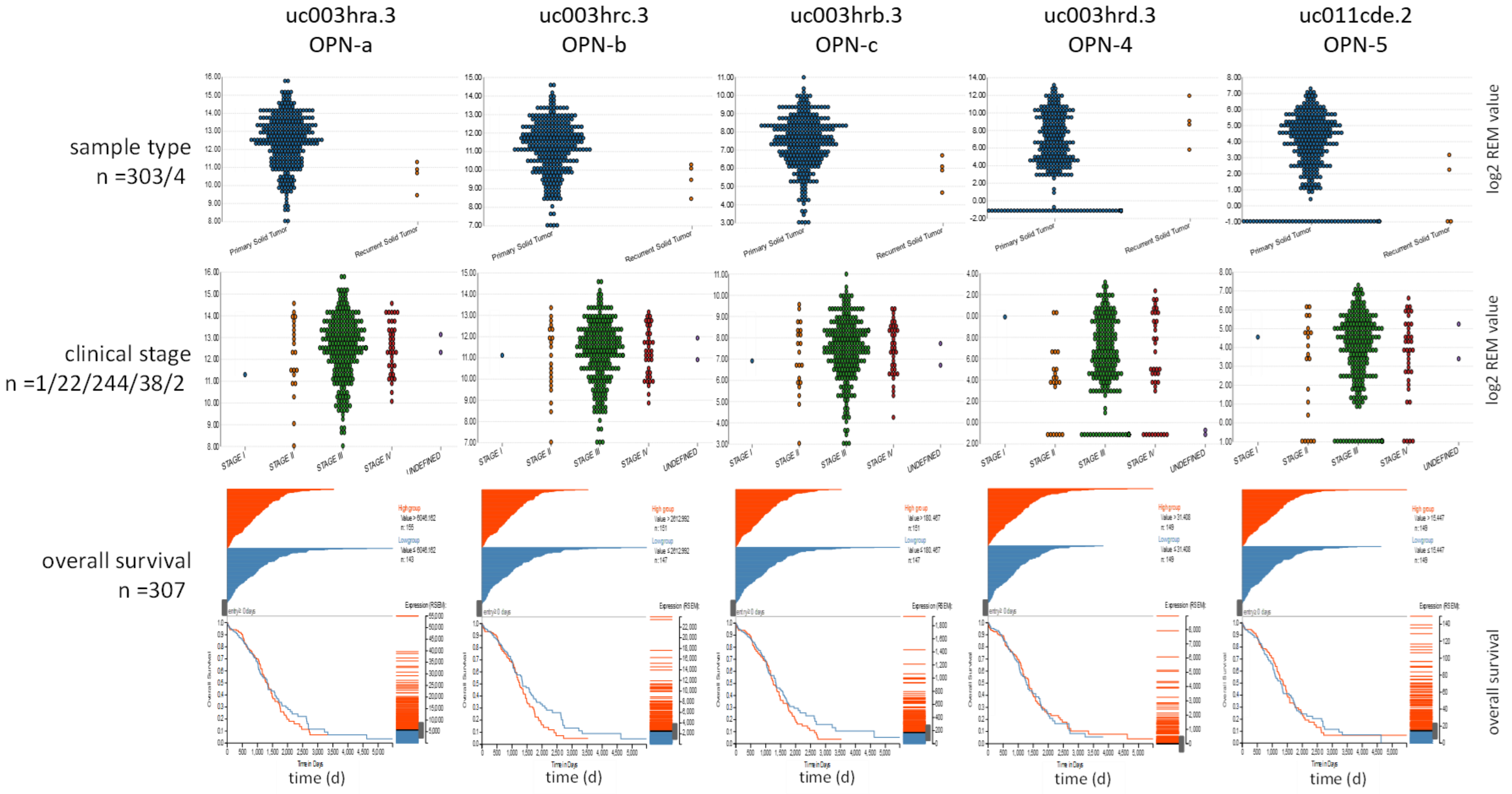

S2.10 cervical cancers

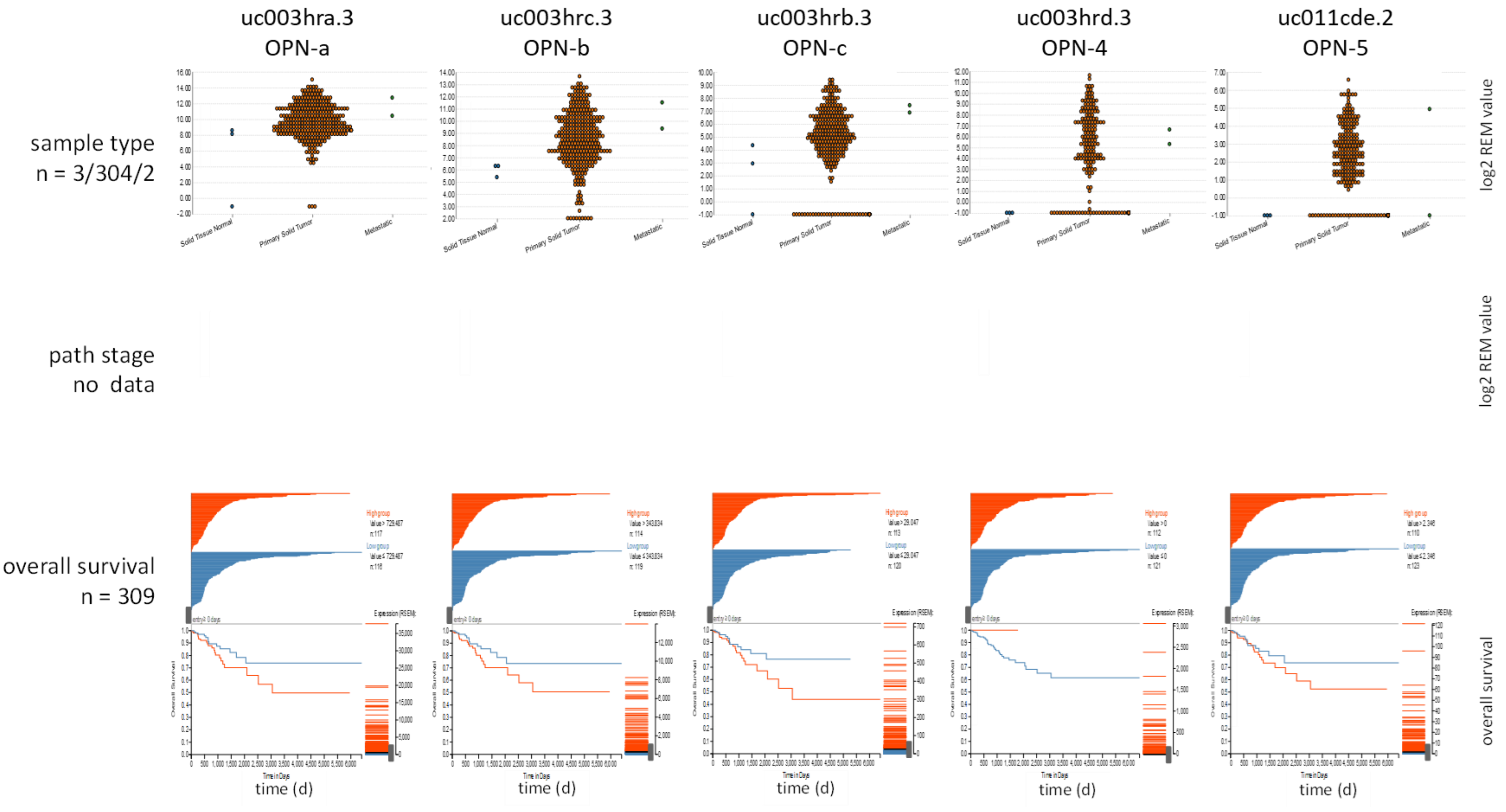

S2.11 uterine corpus endometrial carcinoma

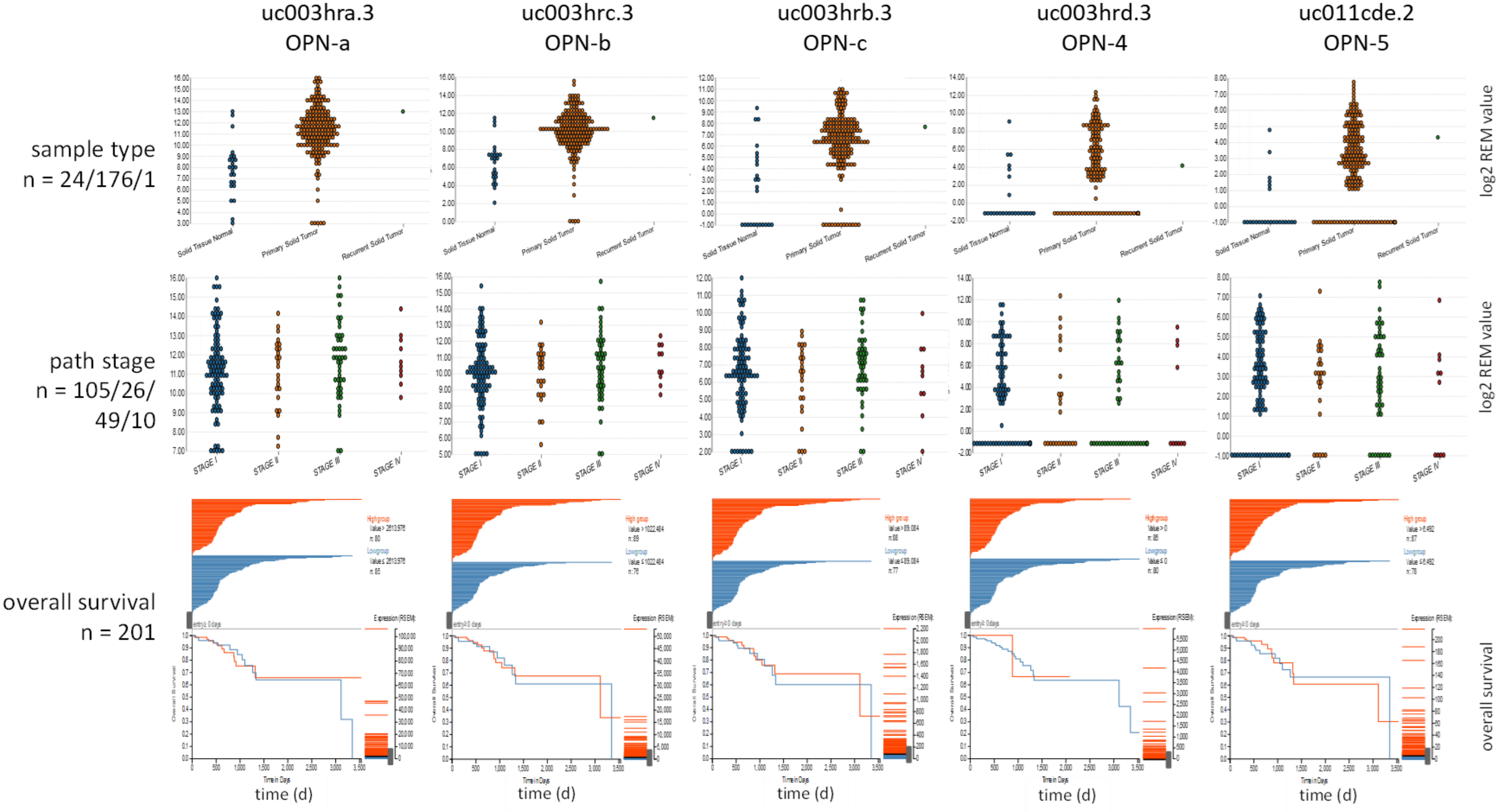

S2.12 uterine carcinosarcoma

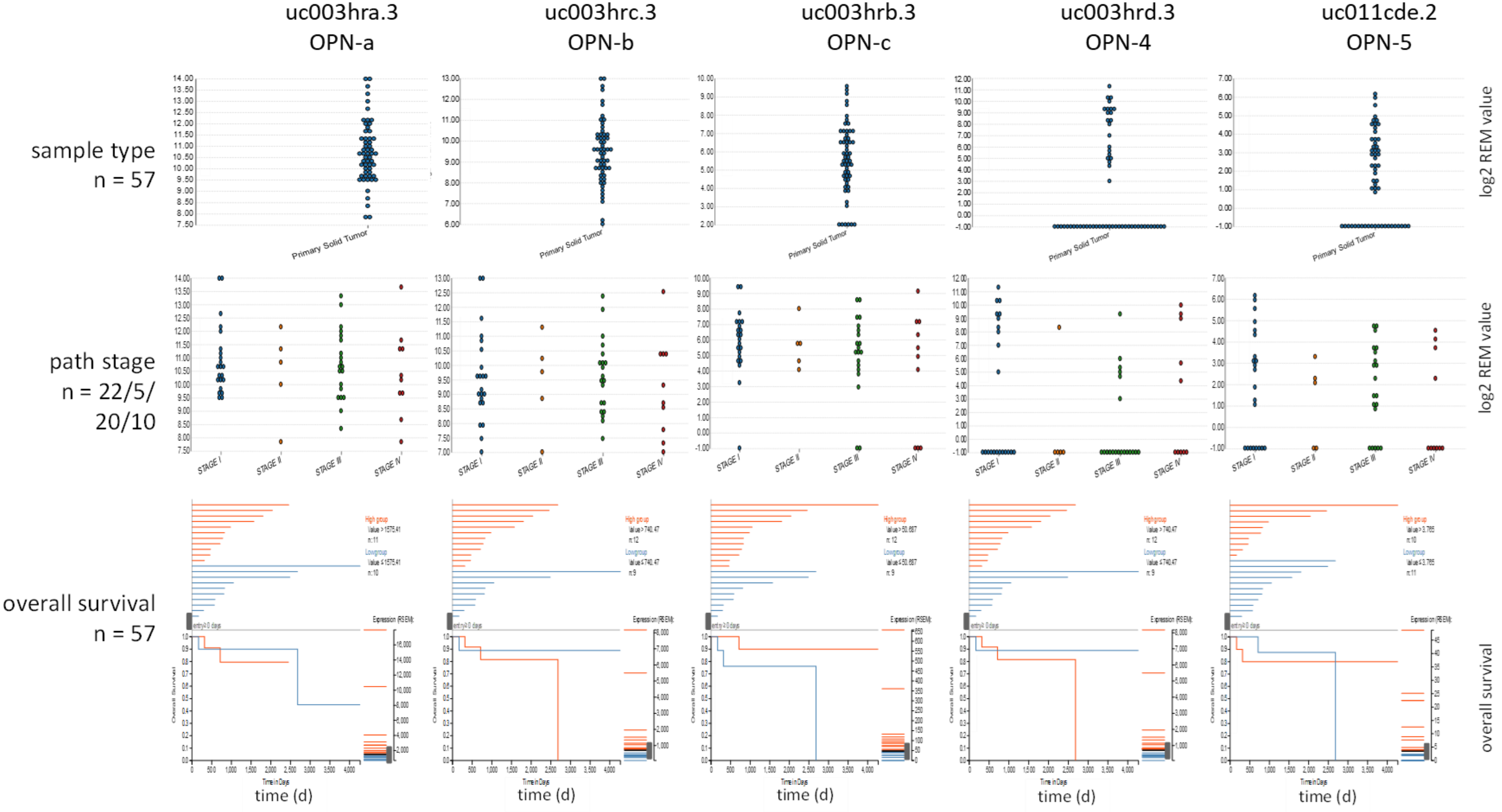

S2.13 breast cancer

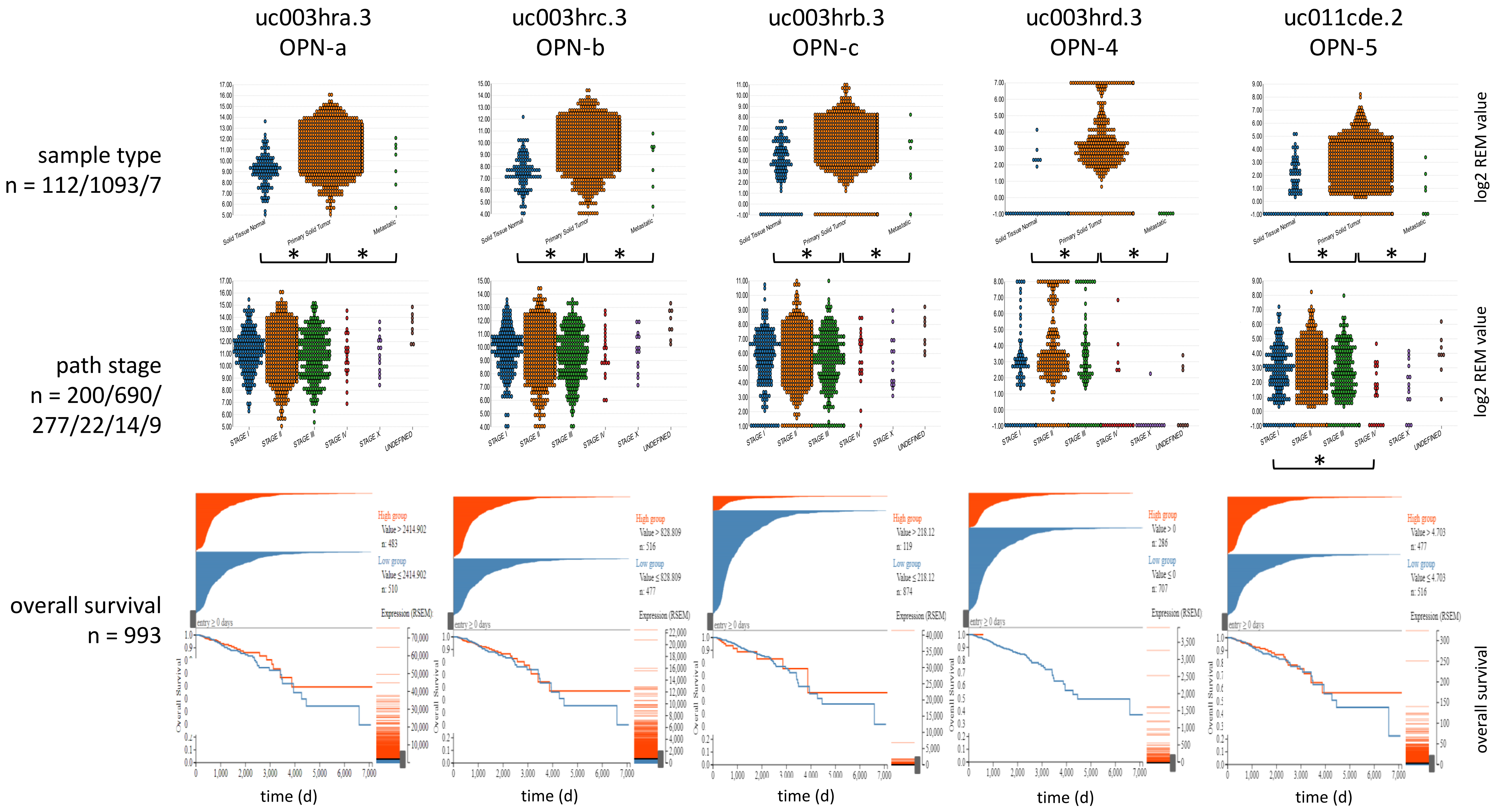

S2.14 esophageal carcinoma

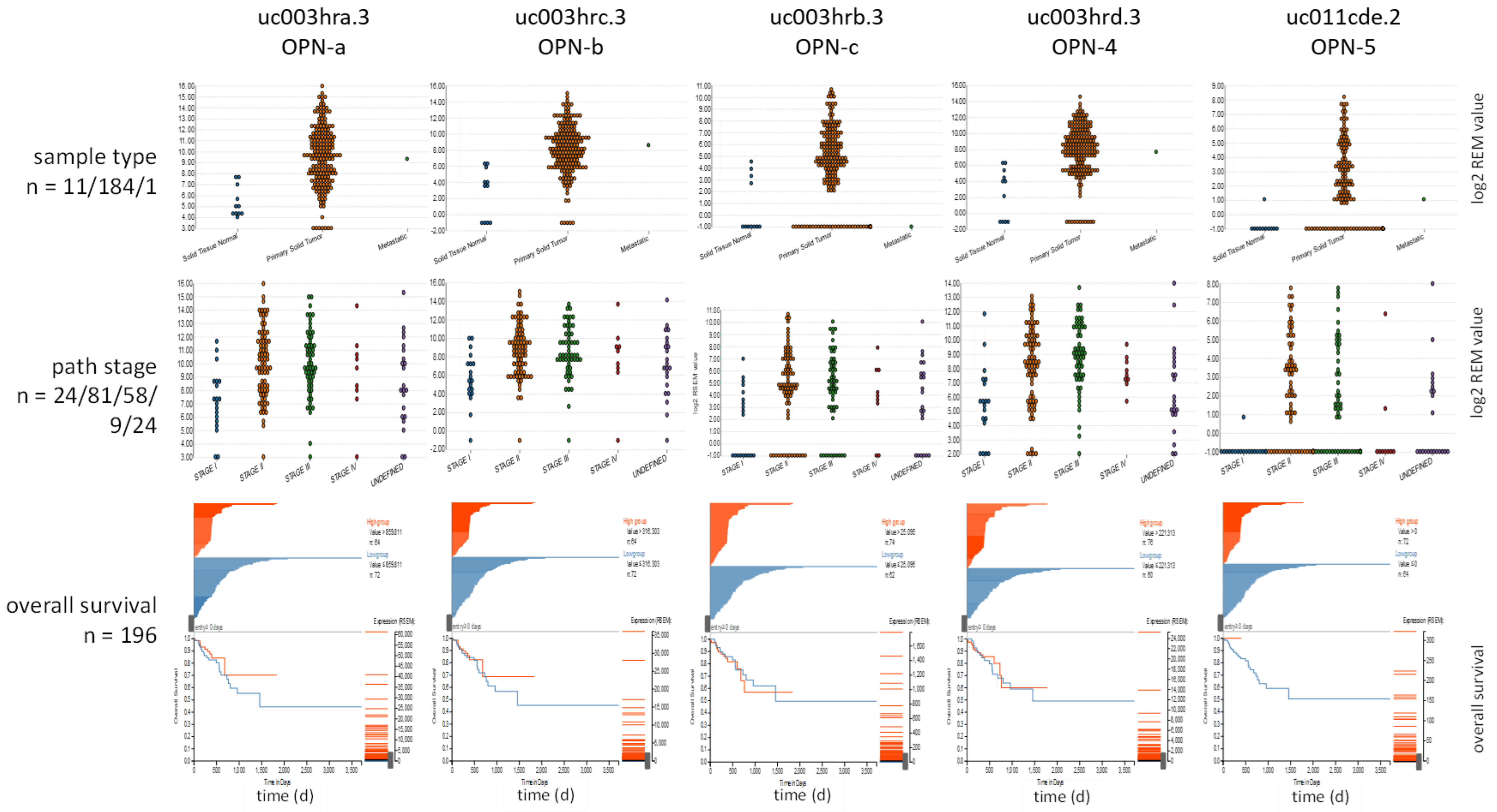

S2.15 stomach adenocarcinoma

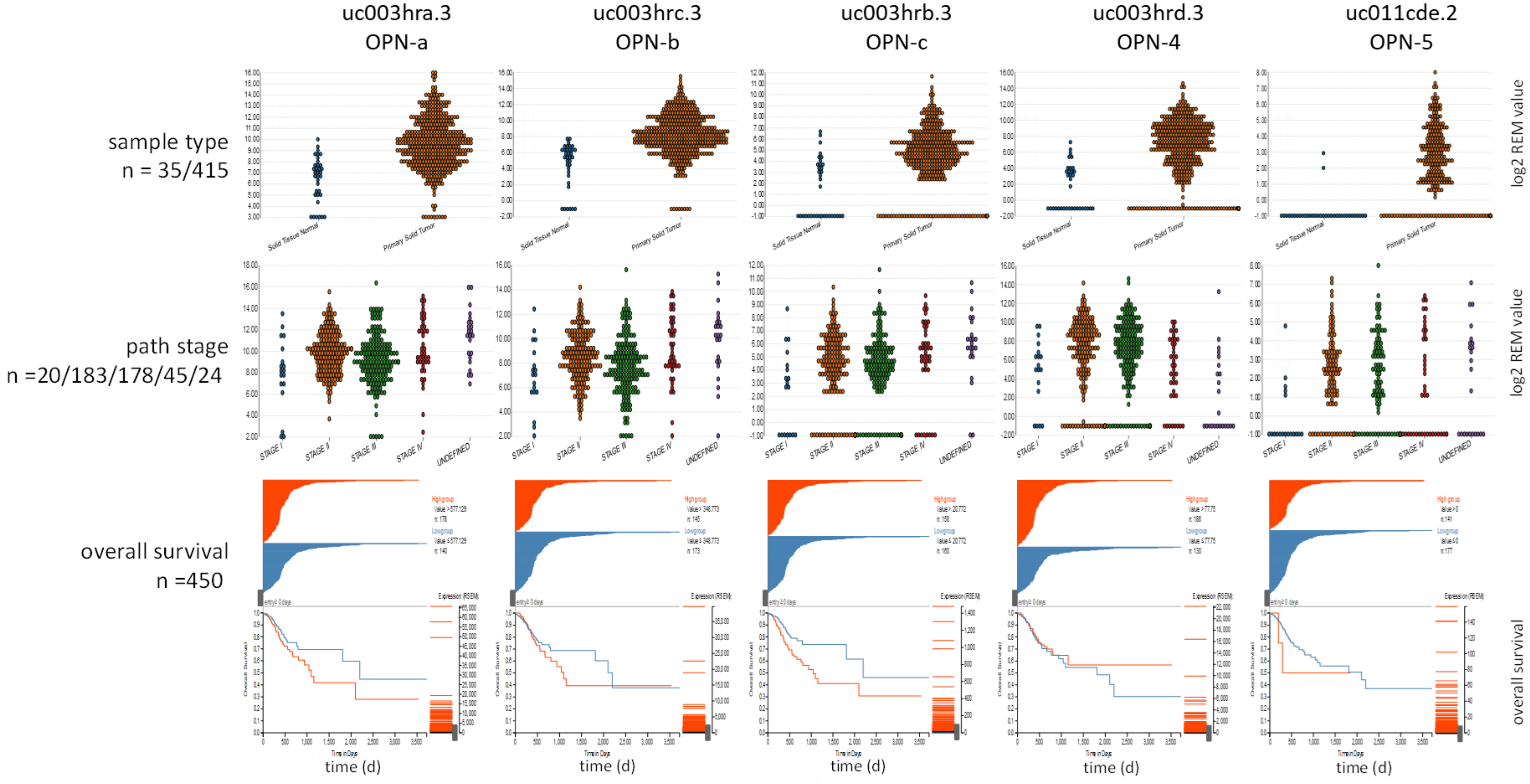

S2.16 colon adenocarcinoma

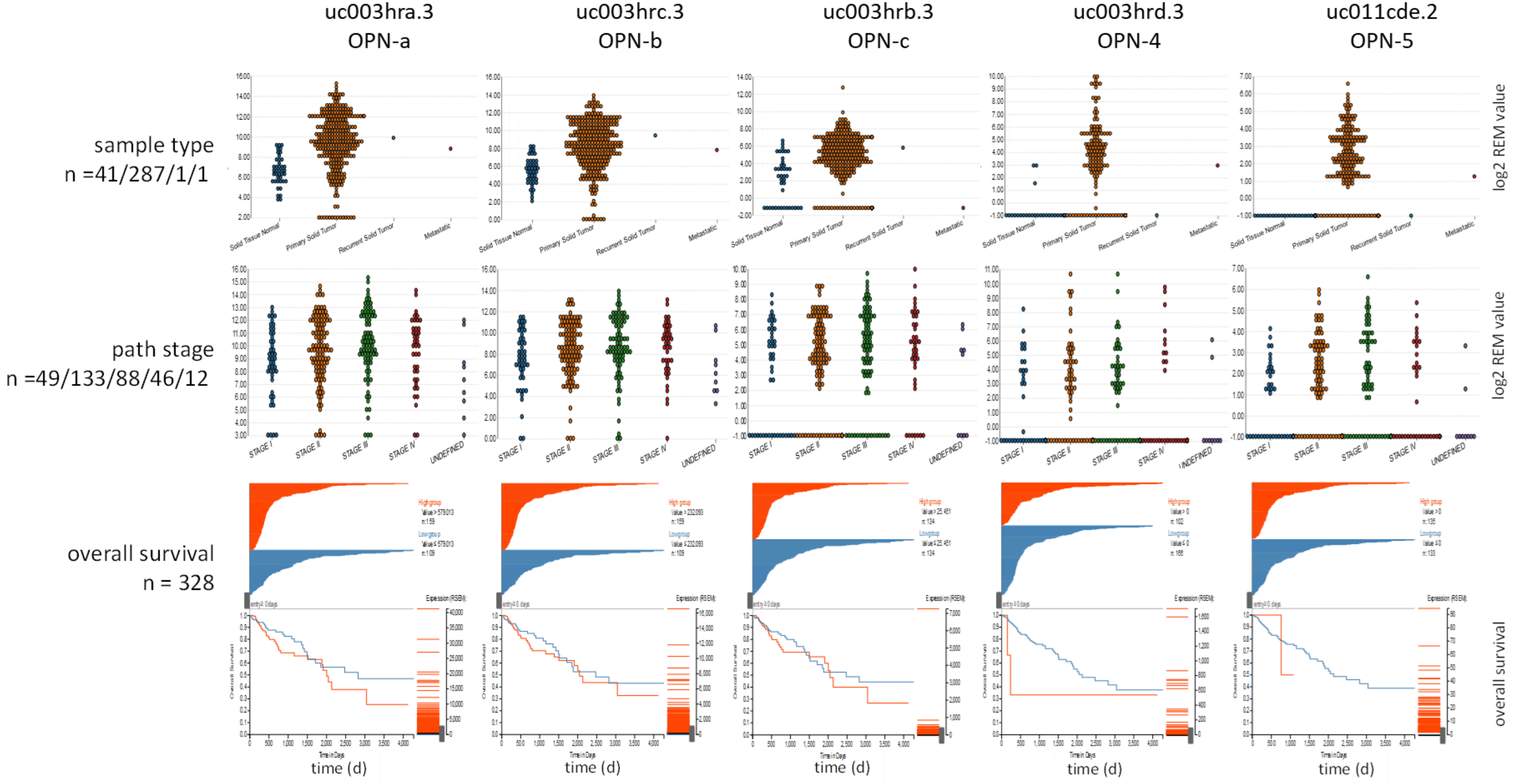

S2.17 rectum adenocarcinoma

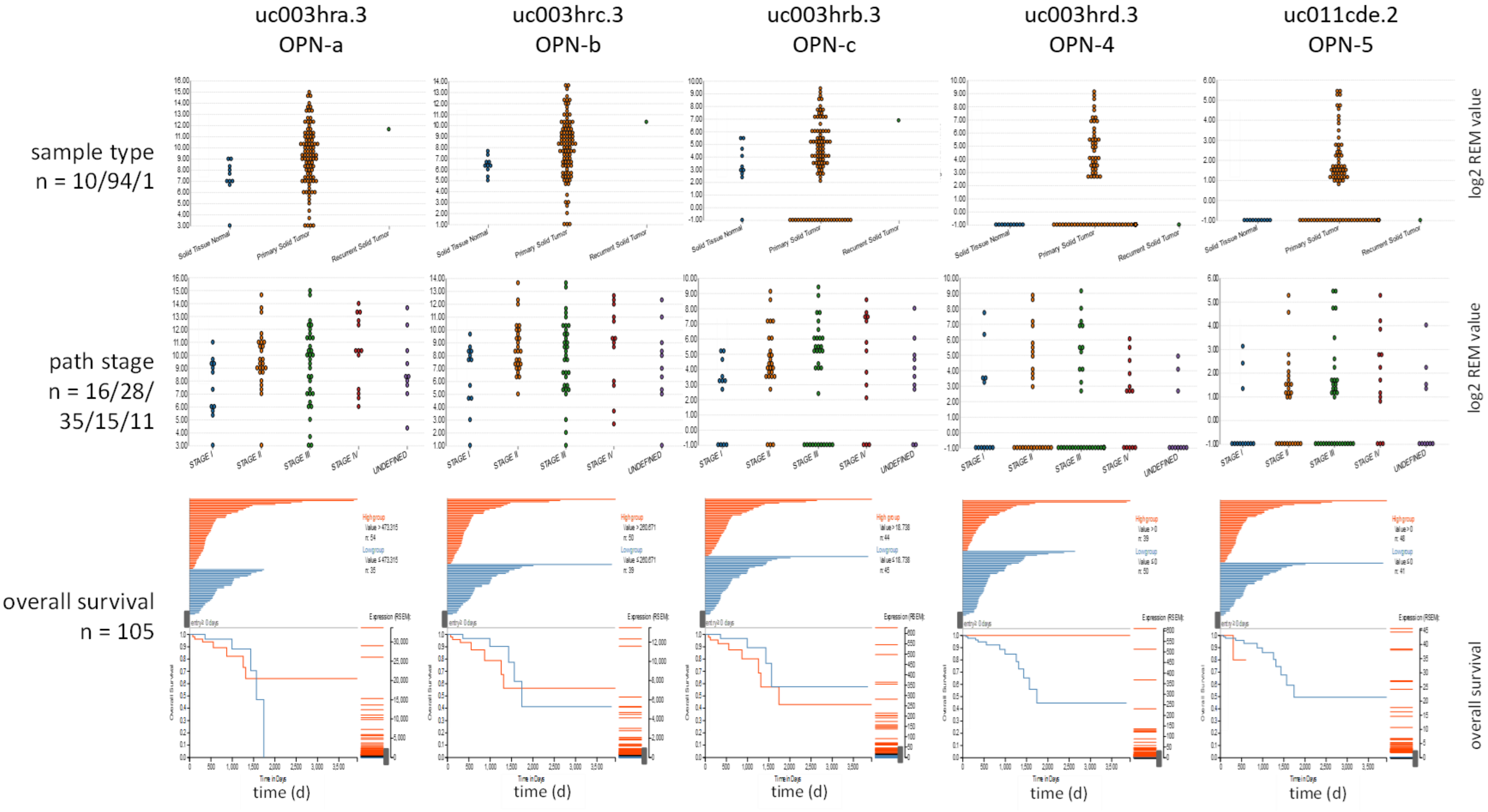

S2.18 chol-angiosarcoma

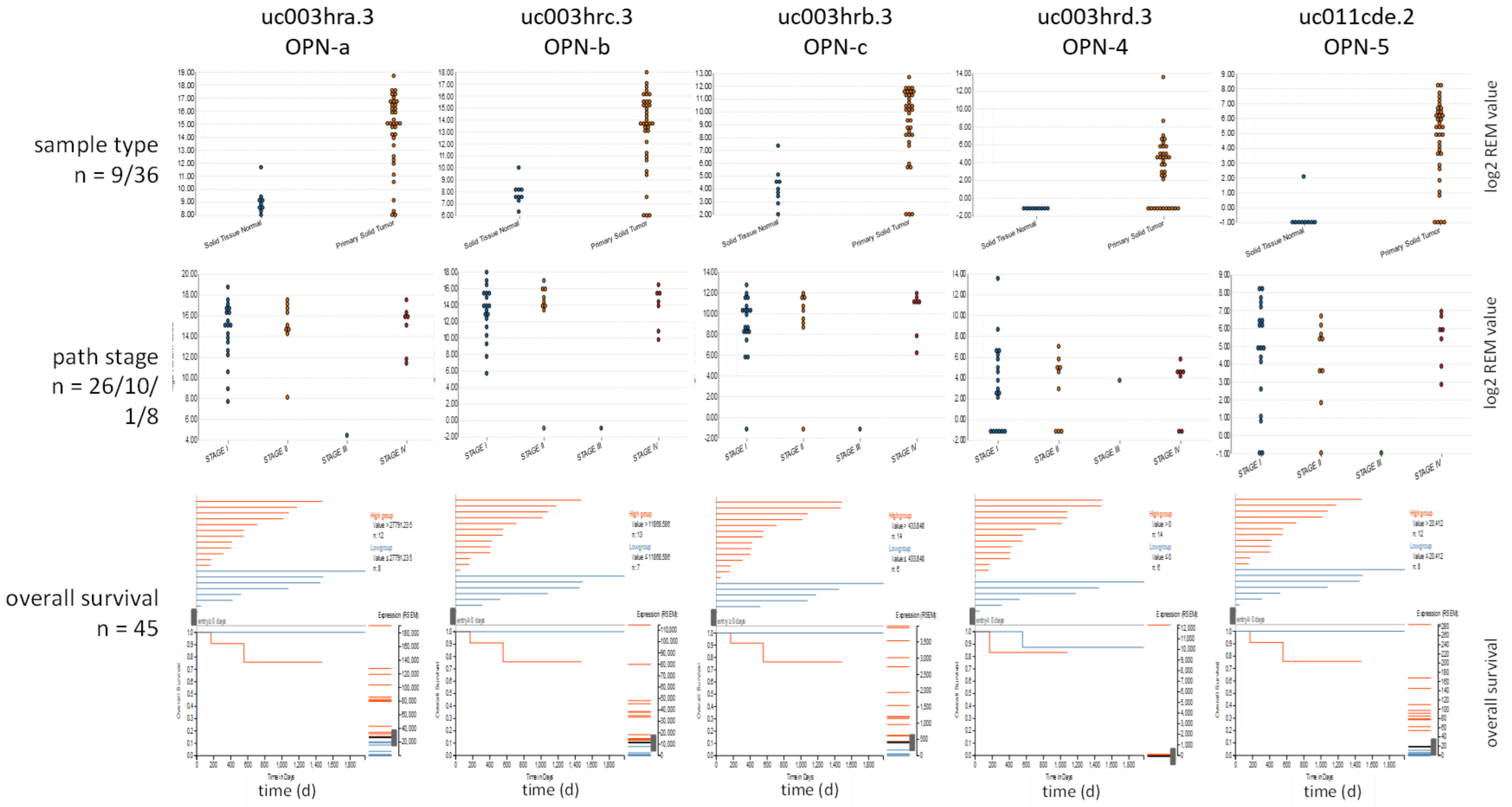

S2.19 liver hepato-cellular carcinoma

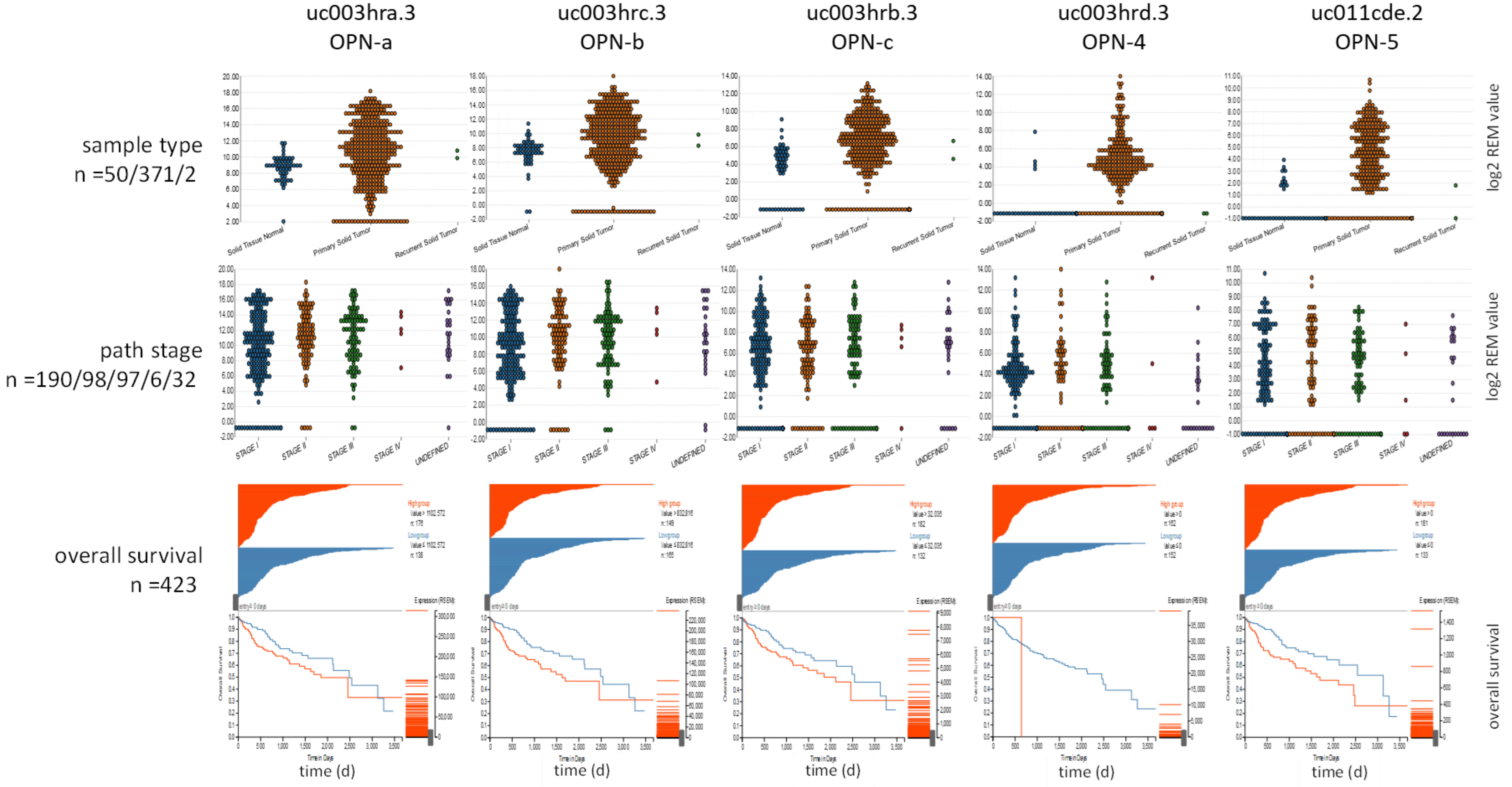

S2.20 pancreatic adenocarcinoma

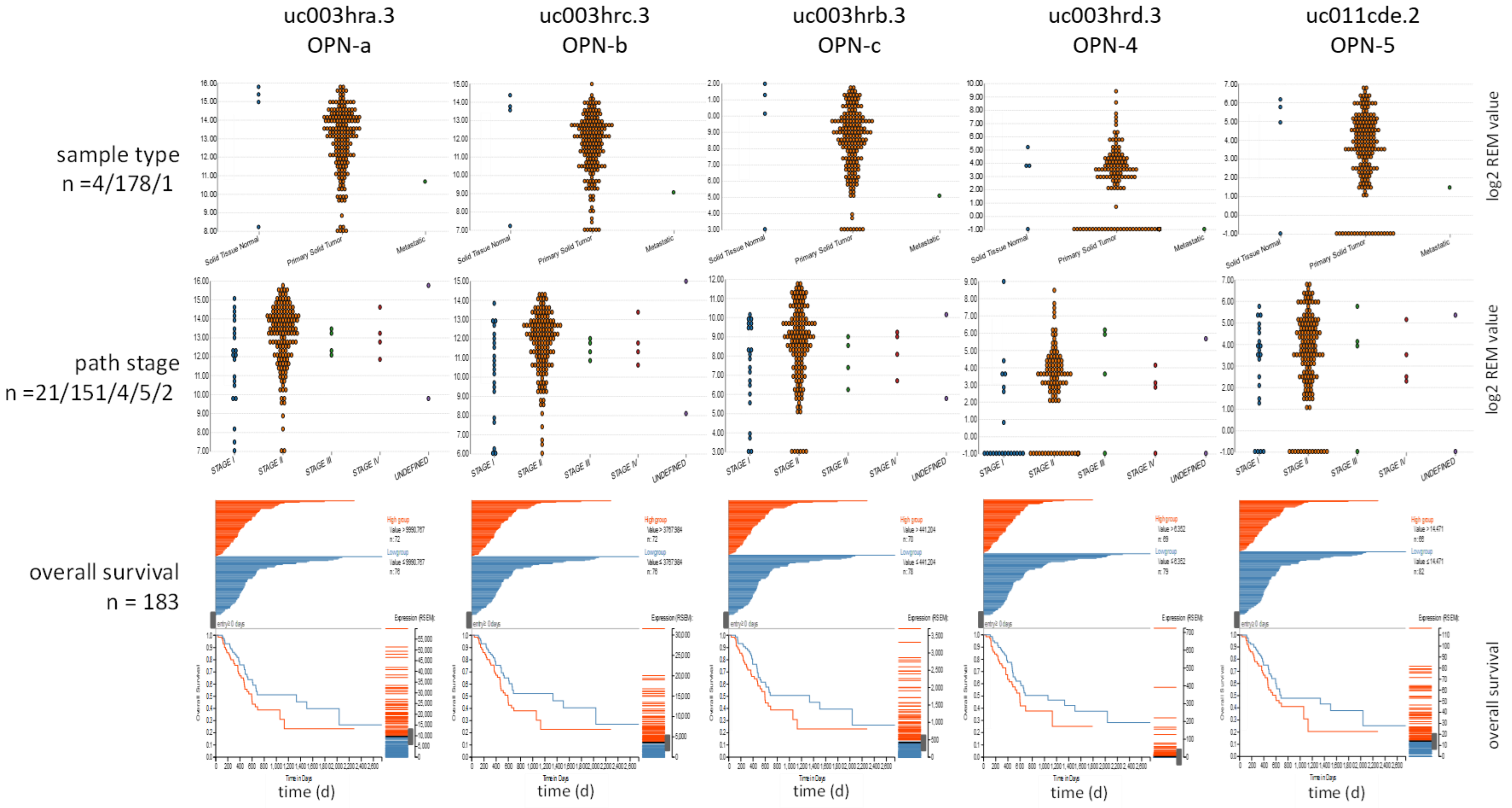

S2.21 lung adenocarcinoma

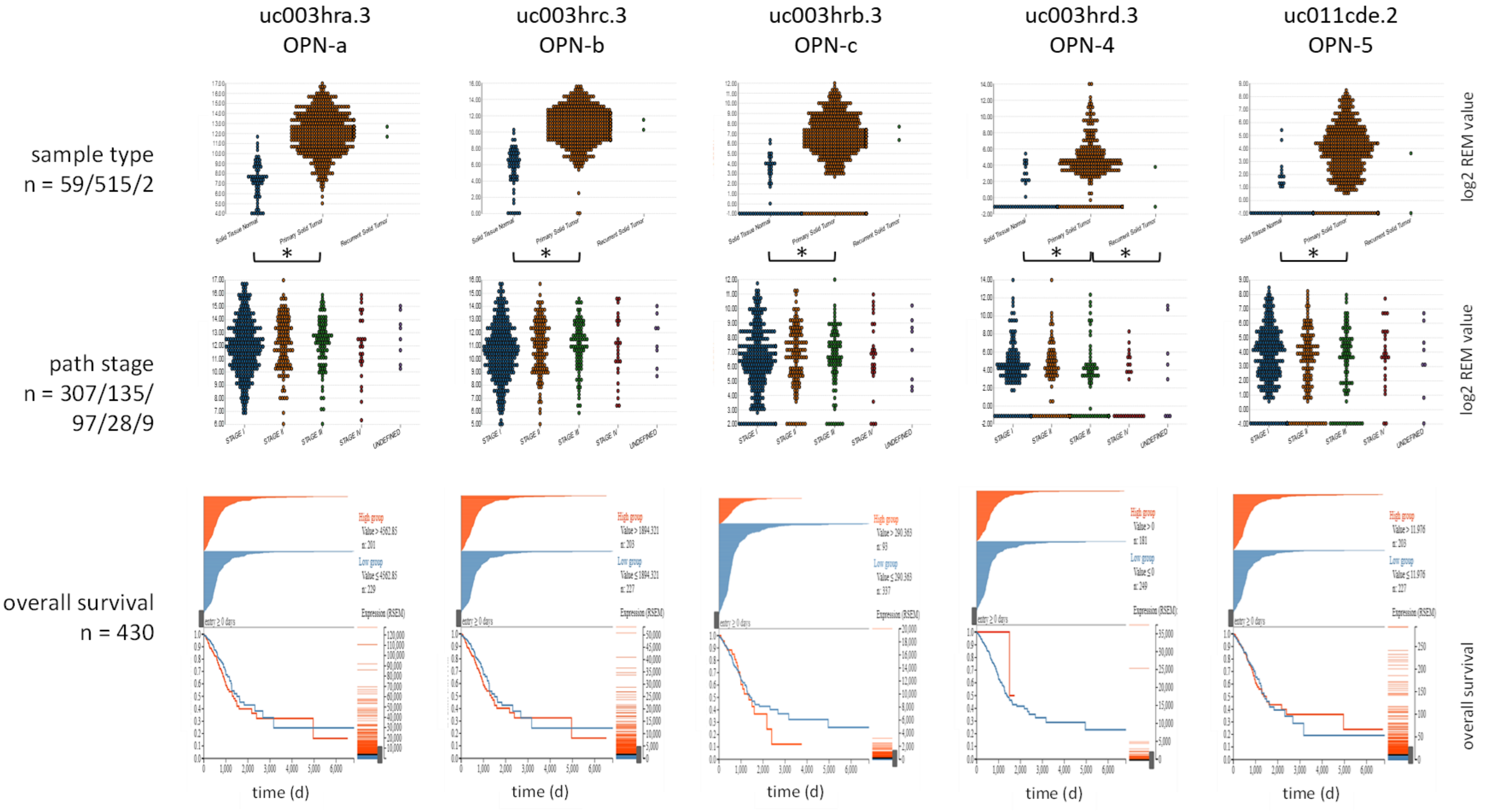

S2.22 lung squamous cell carcinoma

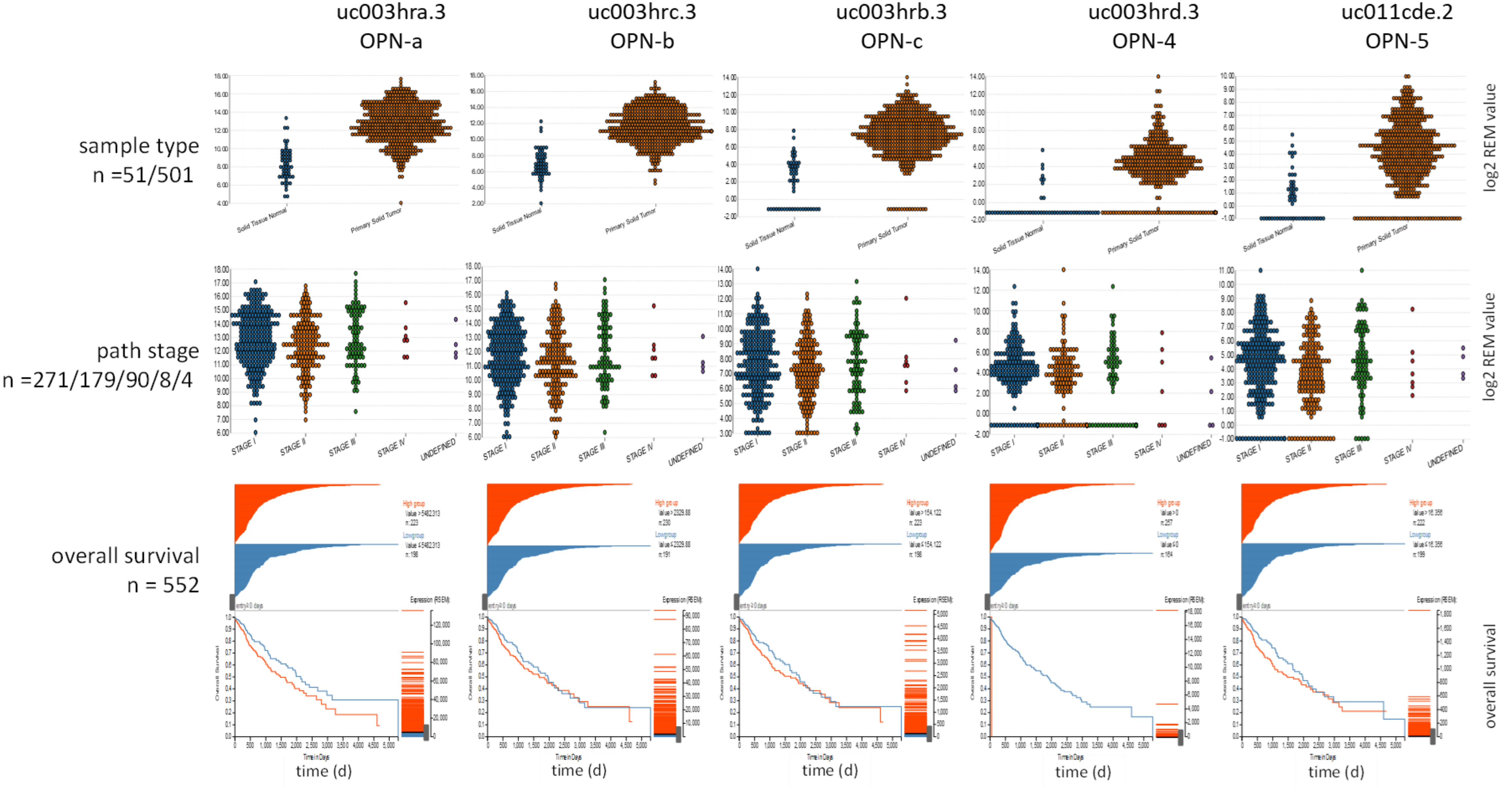

S2.23 mesothelioma

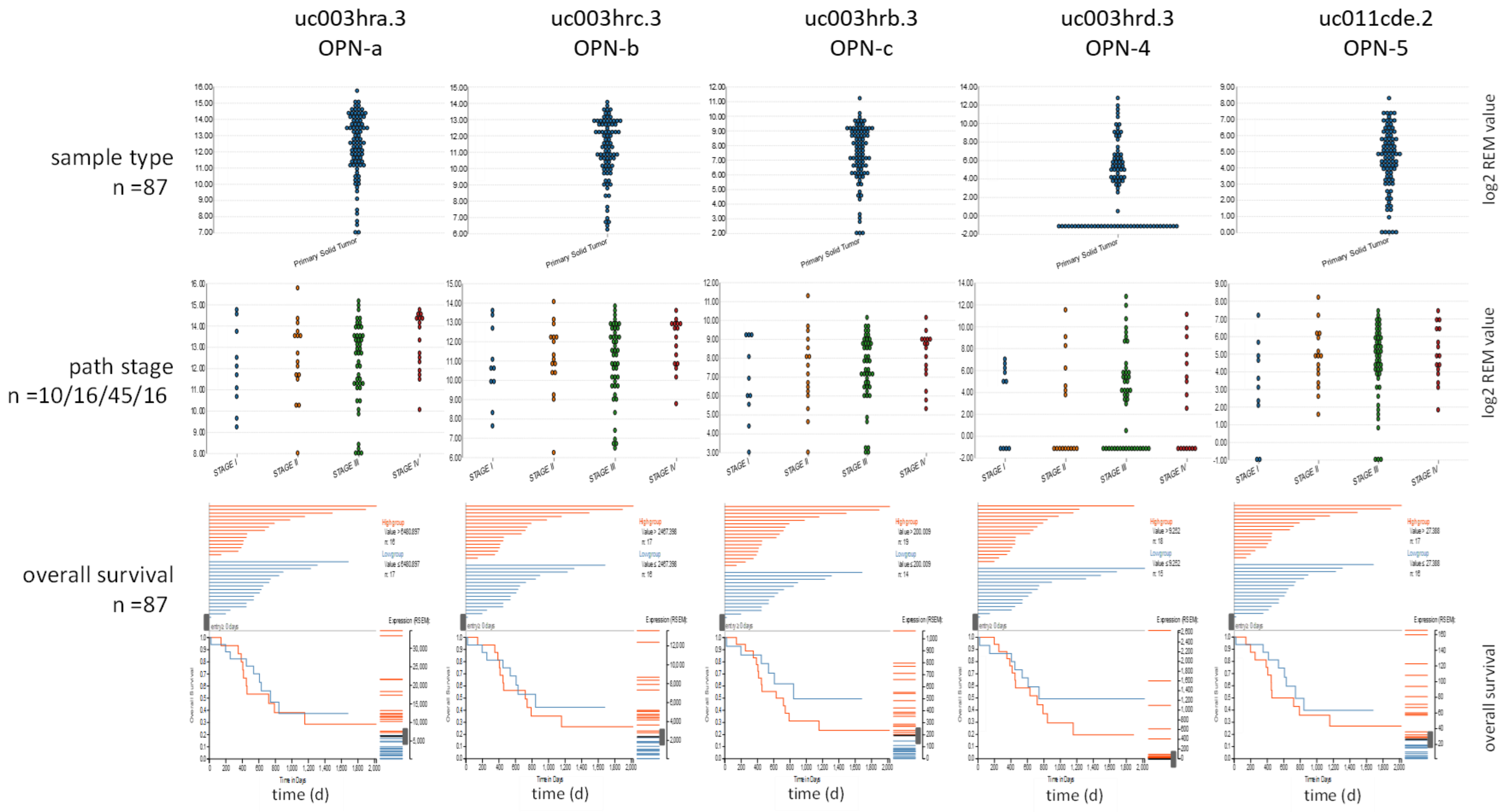

S2.24 thyroid carcinoma

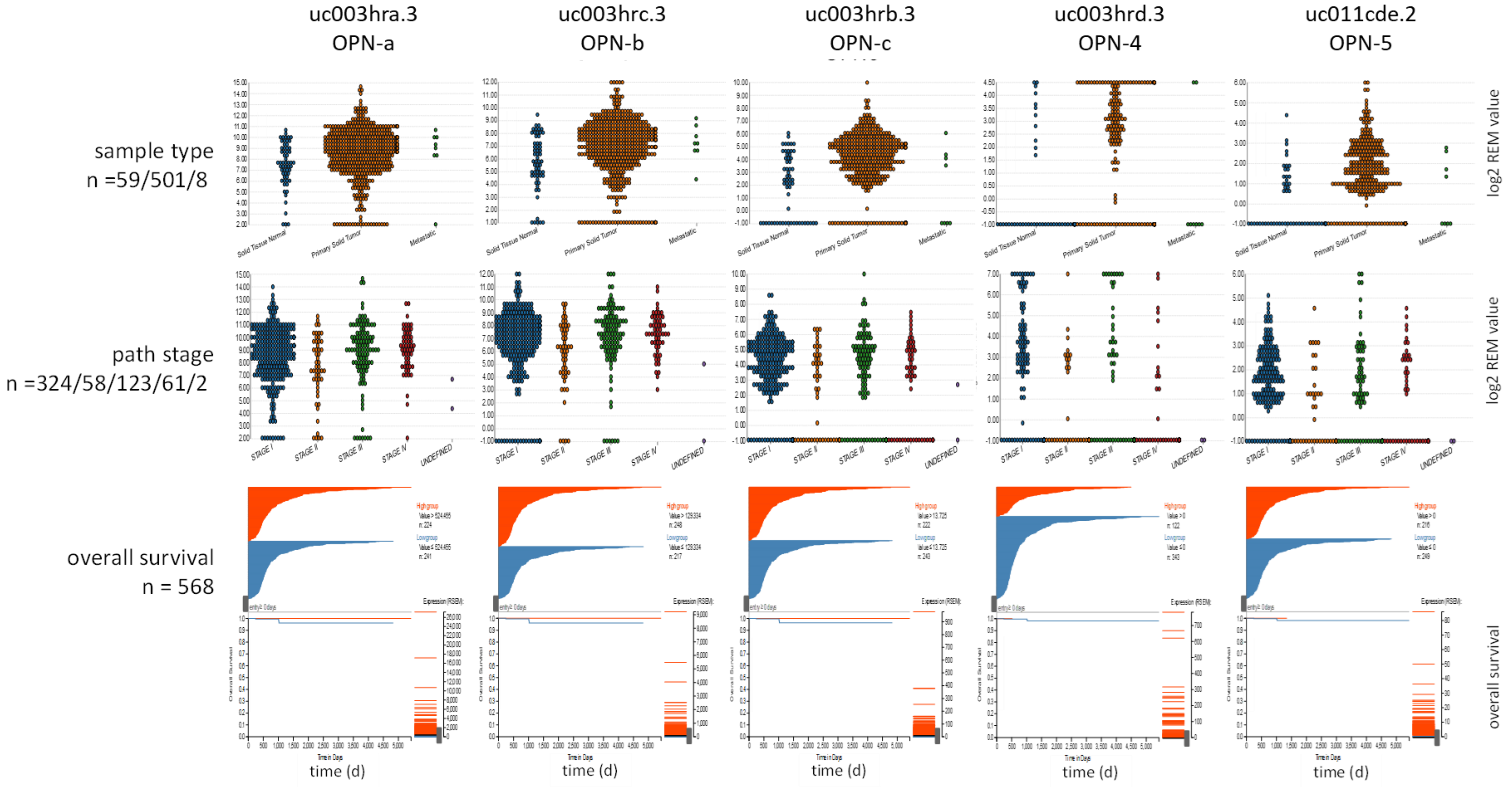

S2.25 thymoma

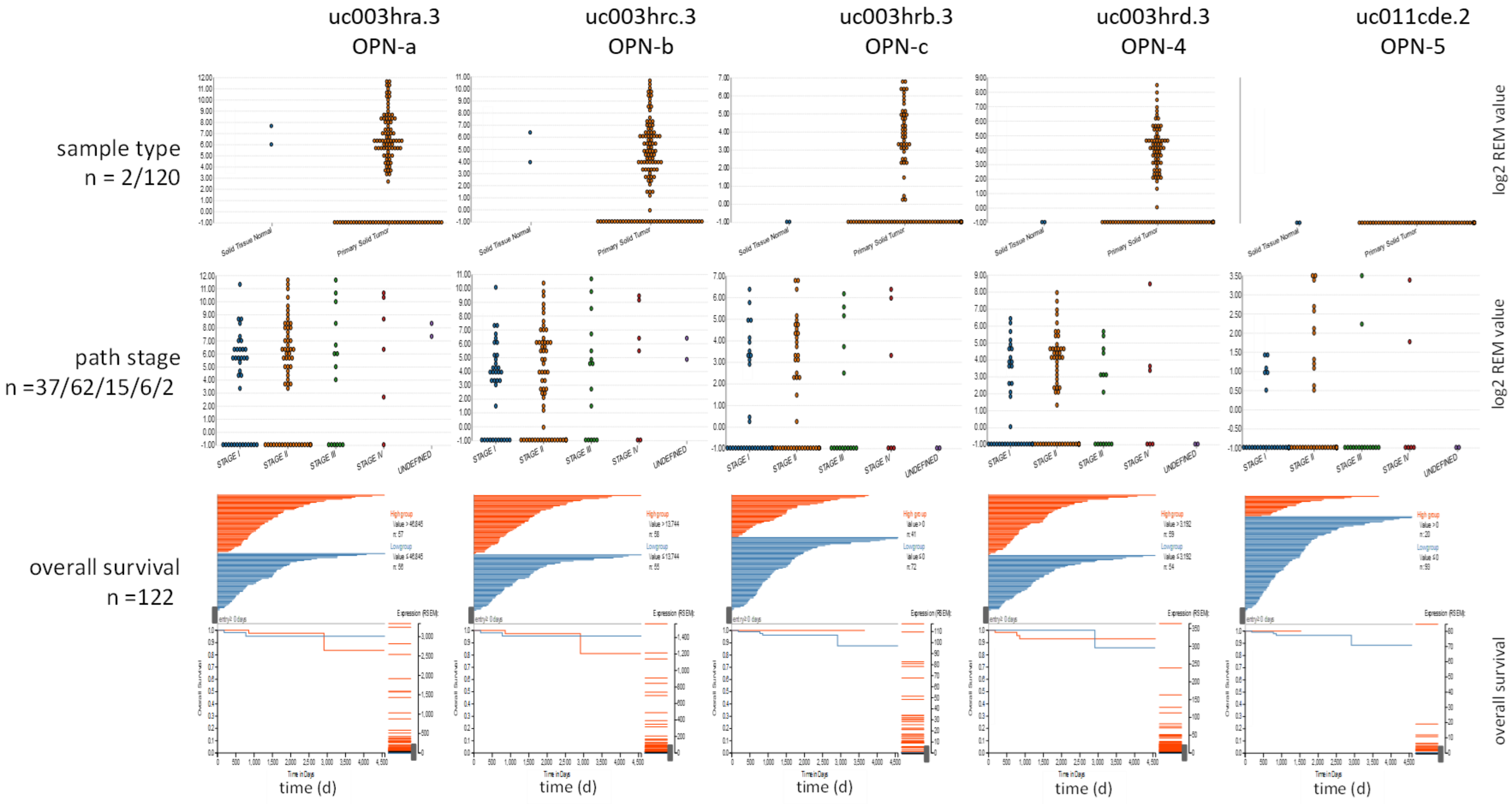

S2.26 diffuse large B-cell lymphoma

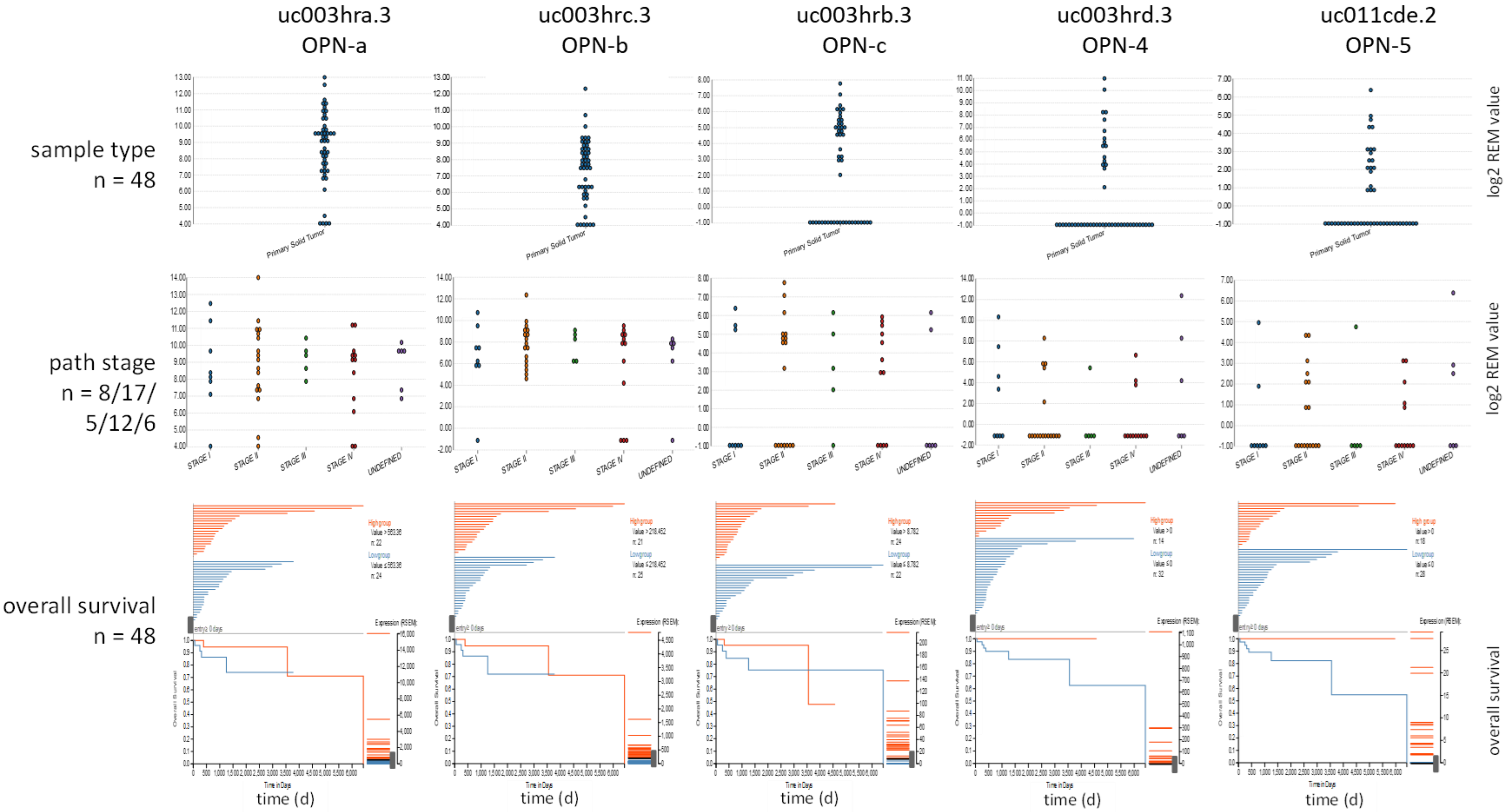

S2.27 acute myeloid leukemia

uc003hra.3 OPN-a	uc003hrc.3 OPN-b	uc003hrb.3 OPN-c	uc003hrd.3 OPN-4	uc011cde.2 OPN-5

sample type
n =173

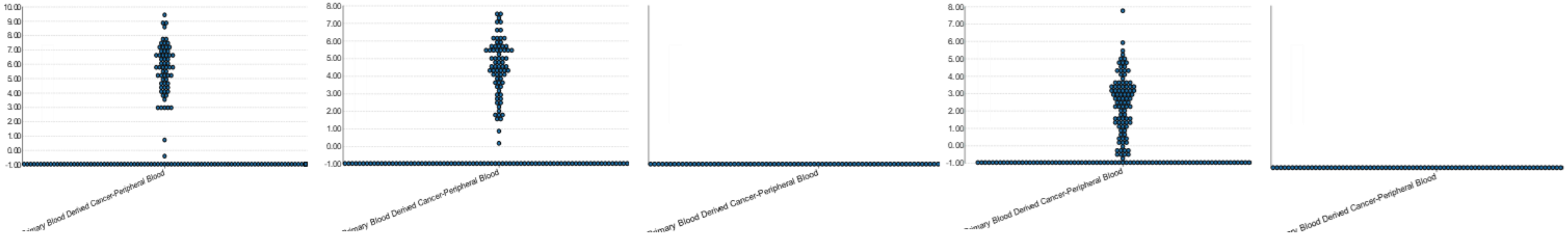

log2 REM value

path stage
no data

log2 REM value

overall survival
no data

overall survival

time (d) time (d) time (d) time (d) time (d)

S2.28 ear and neck cancer

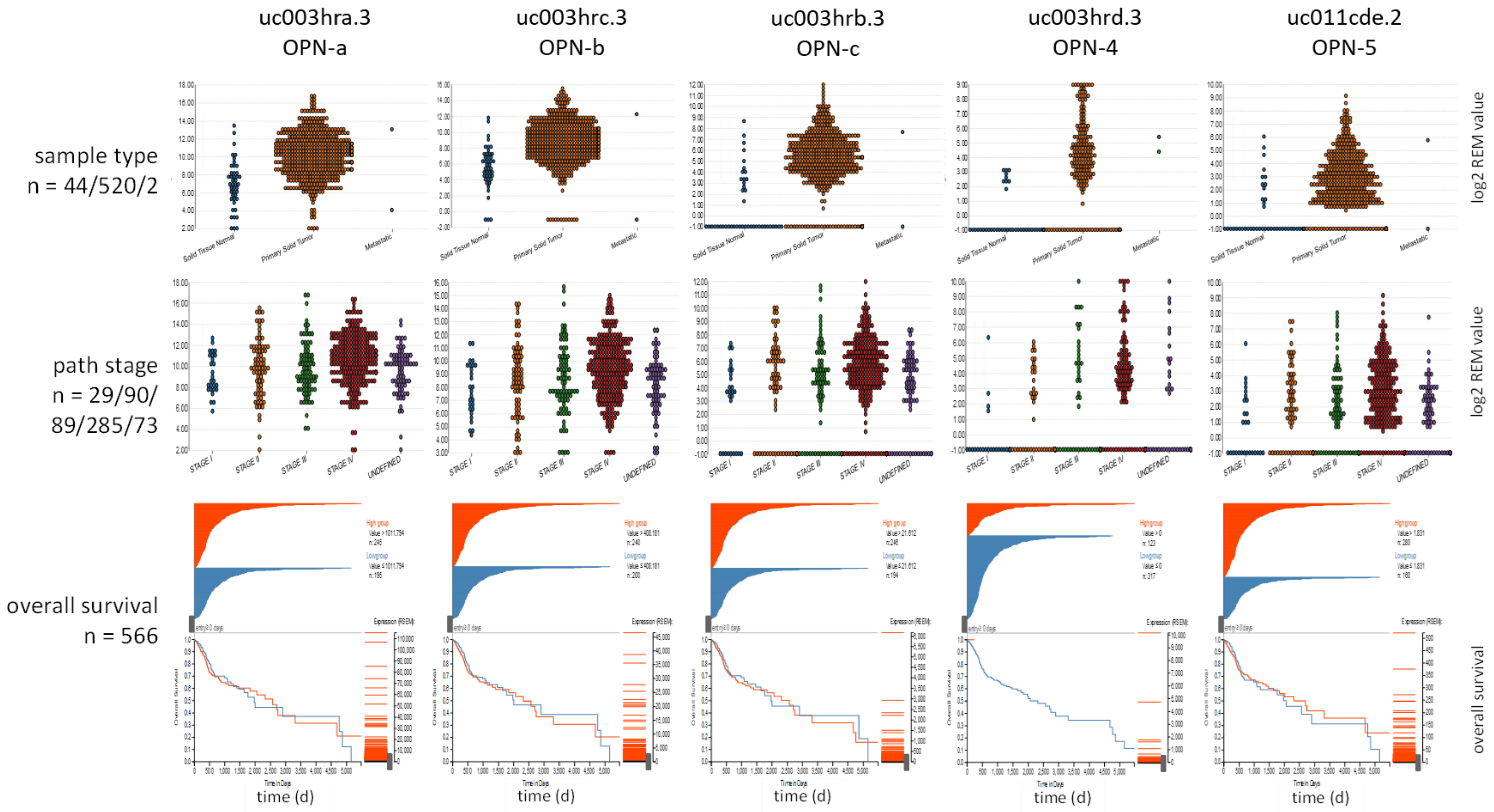

S2.29 glioblastoma

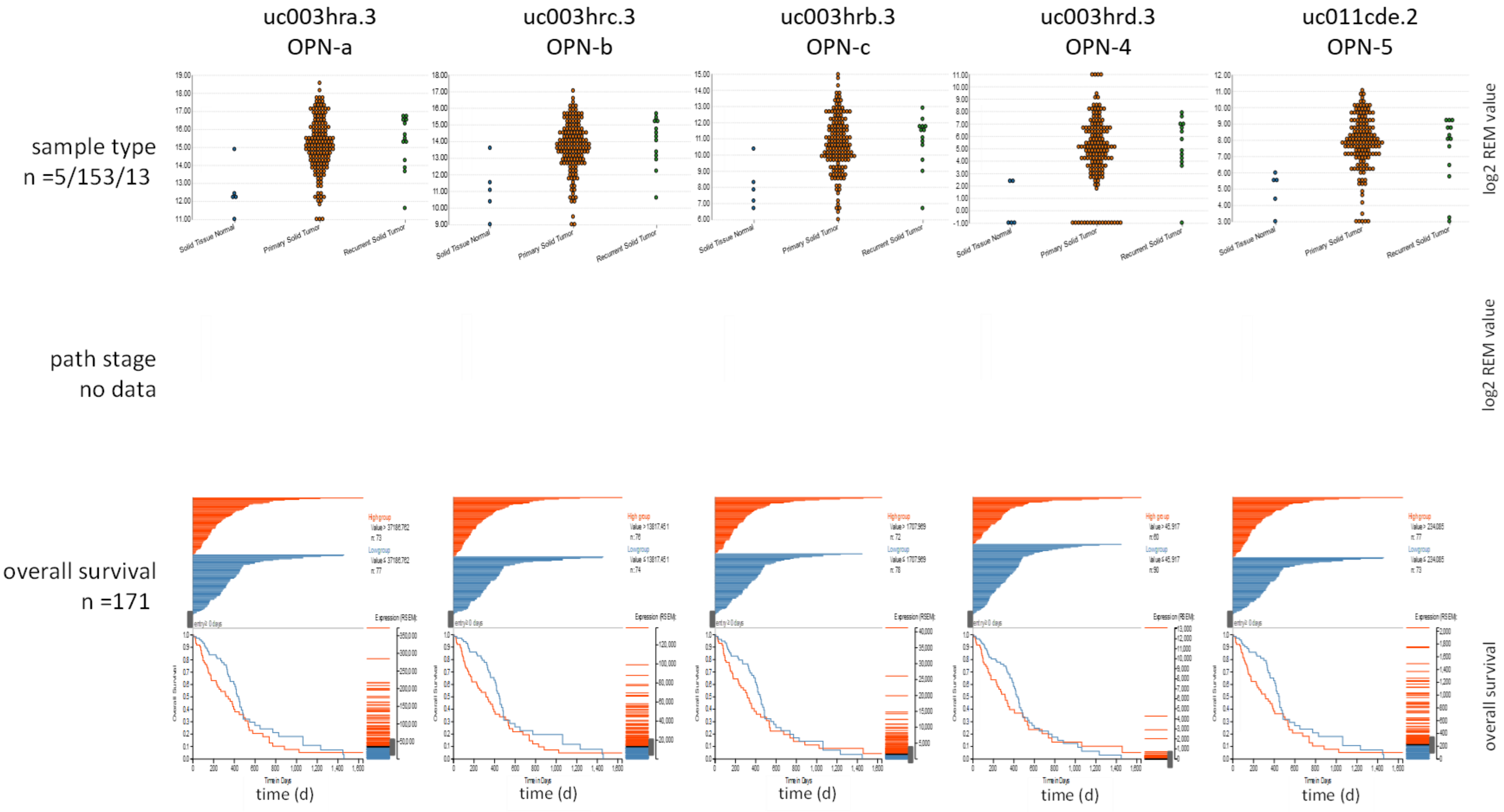

S2.30 lower grade glioma

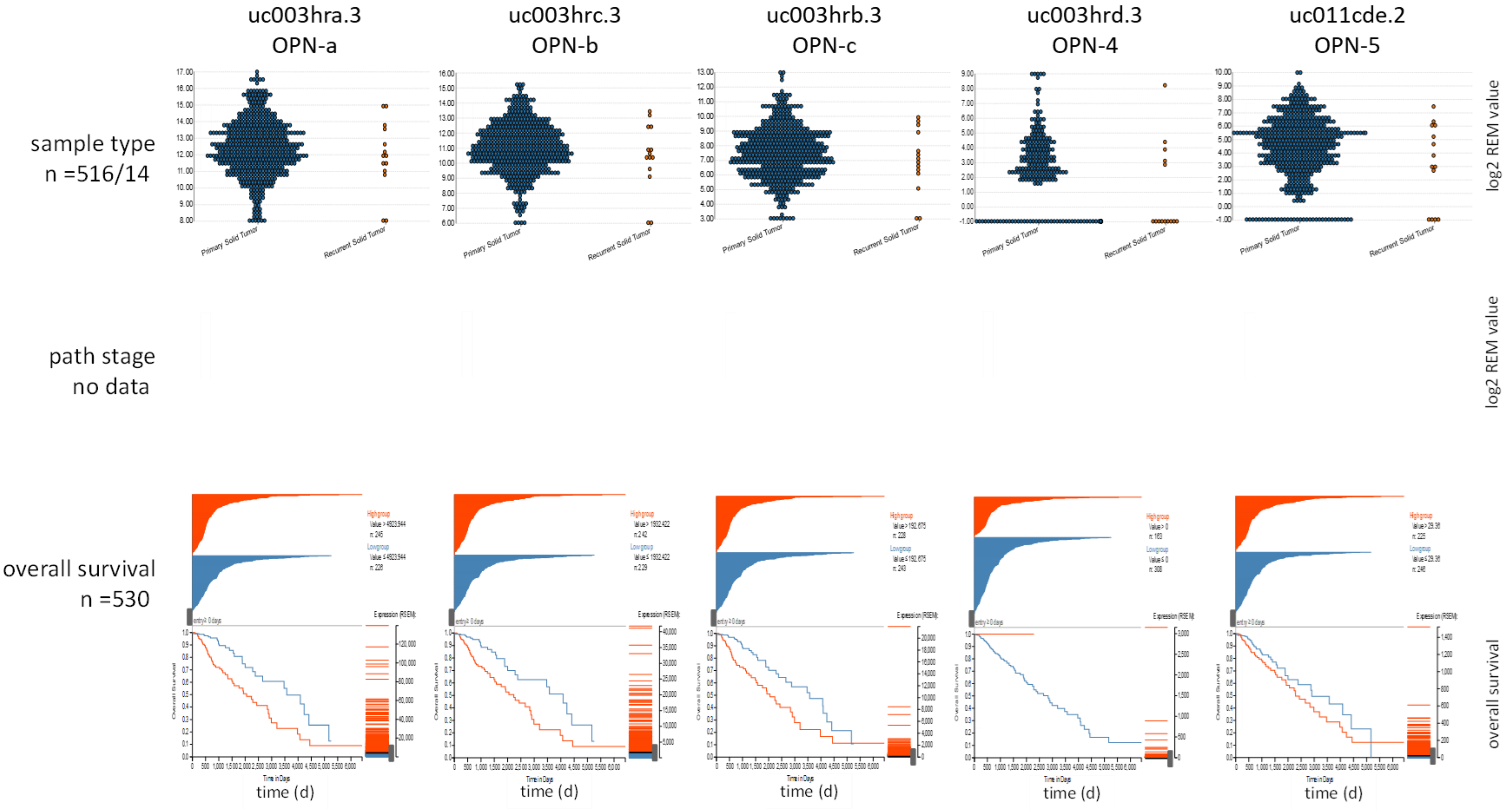

S2.31 cutaneous melanoma

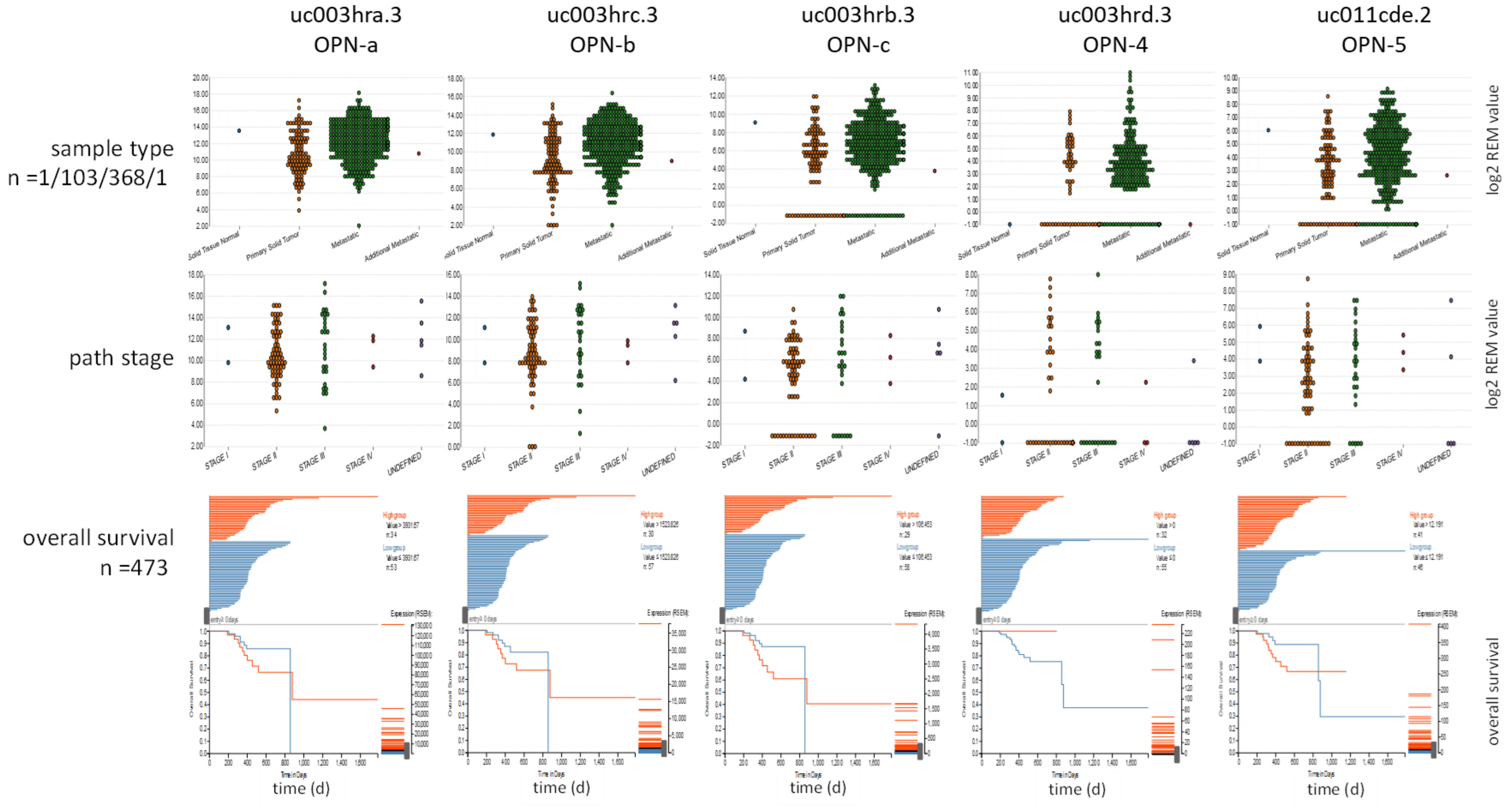

S2.32 uveal melanoma

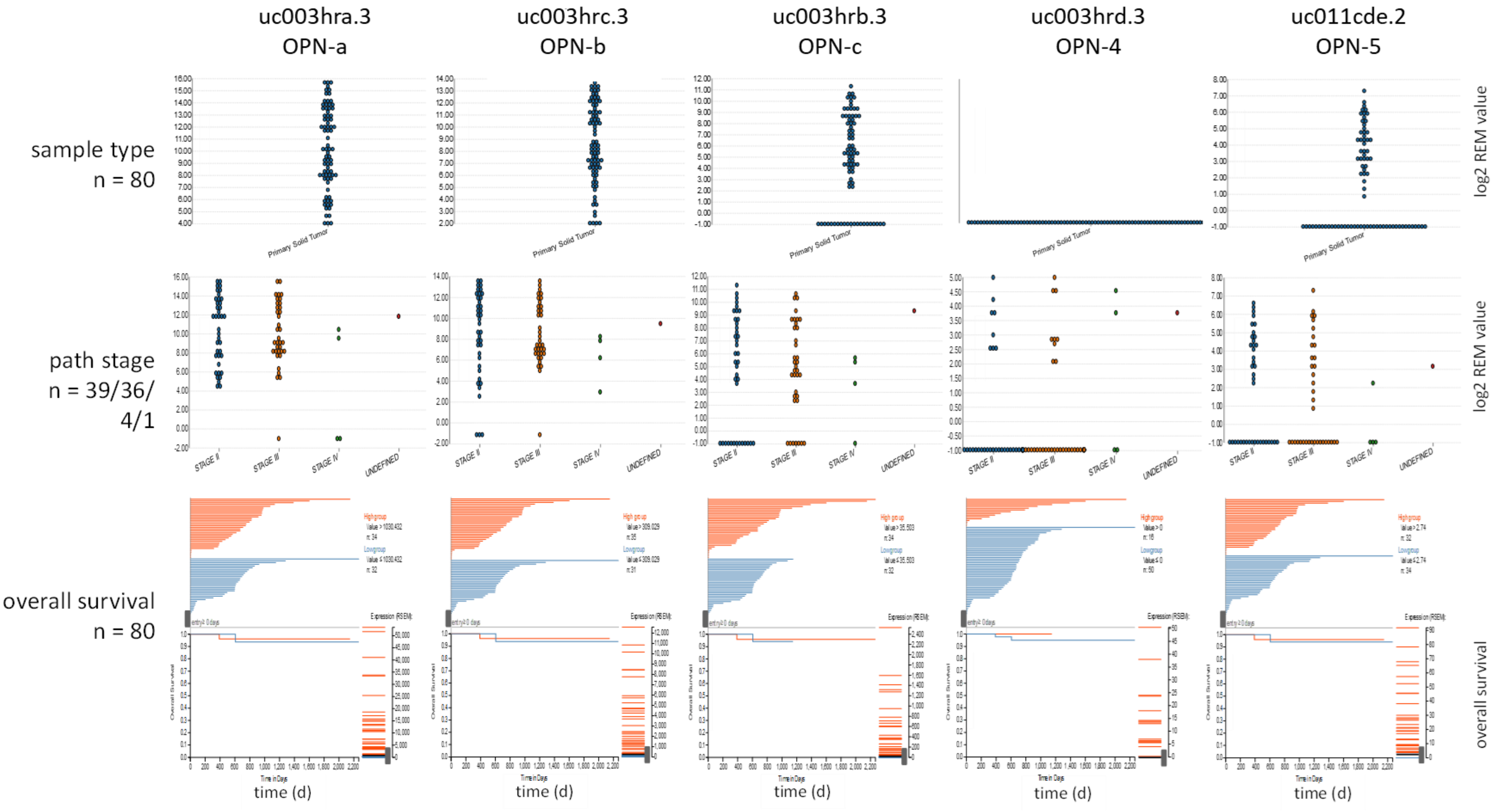

S2.33 sarcoma

uc003hra.3 OPN-a

uc003hrc.3 OPN-b

uc003hrb.3 OPN-c

uc003hrd.3 OPN-4

uc011cde.2 OPN-5

sample type
n = 2/259/3/1

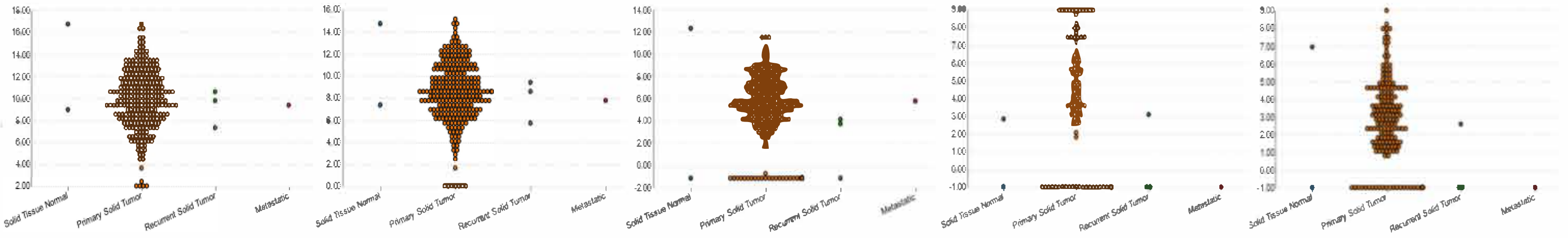

log2 REM value

path stage
no data

log2 REM value

overall survival
n =265

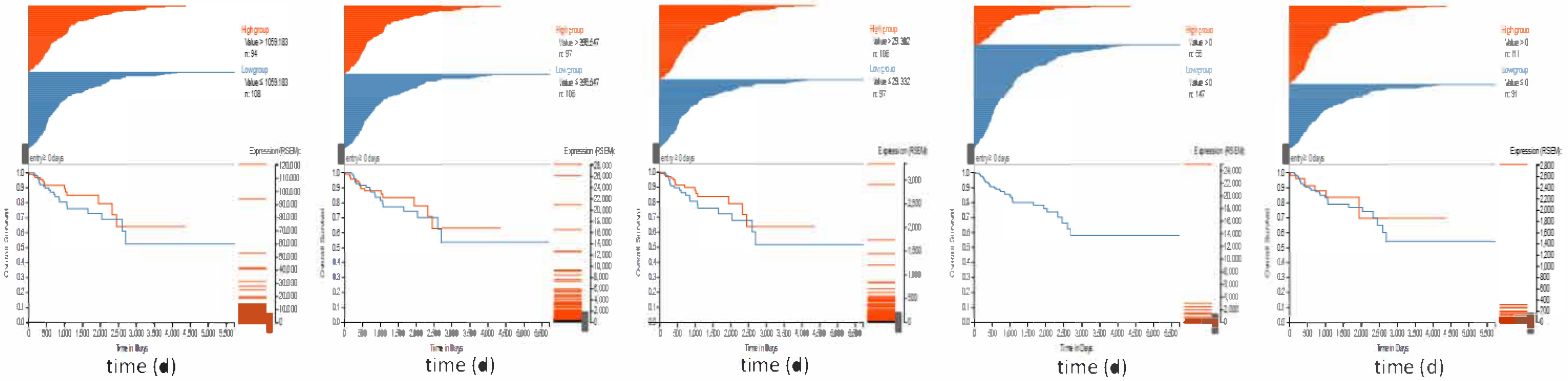

overall survival
